# Electronic Decision Support for Deprescribing in Older Adults Living in Long-Term Care: A Stepped-Wedge Cluster Randomized Trial

**DOI:** 10.1001/jamanetworkopen.2025.12931

**Published:** 2025-05-30

**Authors:** Emily G. McDonald, Justine L. Estey, Cody Davenport, Émilie Bortolussi-Courval, Jeffrey Gaudet, Pierre Philippe Wilson Registe, Todd C. Lee, Carole Goodine

**Affiliations:** 1 Division of General Internal Medicine, Department of Medicine, McGill University Health Centre, Montreal, Quebec, Canada; 2 Division of Experimental Medicine, Department of Medicine, McGill University, Montreal, Quebec, Canada; 3 The Centre for Innovation and Research in Aging, Fredericton, New Brunswick, Canada; 4 Vitalité Health Network, Moncton, New Brunswick, Canada; 5 Maritime SPOR SUPPORT Unit, Halifax, Nova Scotia, Canada; 6 Université de Sherbrooke, Montreal, Québec, Canada; 7 Centre de Formation Médicale du Nouveau Brunswick, Moncton, New Brunswick, Canada; 8 Division of Infectious Diseases, Department of Medicine, McGill University Health Centre, Montreal, Quebec, Canada; 9 Horizon Health Network, Pharmacy Services, Doctor Everett Chalmers Regional Hospital, Fredericton, New Brunswick, Canada

## Abstract

**Importance:**

Potentially inappropriate prescribing (PIP) is common, costly, and harmful. Deprescribing potentially inappropriate medications (PIMs) is a priority for improving the health outcomes of older adults. PIP is especially common in long-term care homes, with up to 88% of residents affected.

**Objective:**

To assess the efficacy of electronic decision support for deprescribing in long-term care.

**Design, Setting, and Participants:**

This stepped-wedge cluster randomized trial took place from August 1, 2021, to October 31, 2022, during the COVID-19 pandemic. The study assessed older adults residing in 1 of 5 long-term care homes in New Brunswick, Canada, at the start of the study who were prescribed 1 or more PIMs. The 5 long-term care homes were divided into 3 clusters. All clusters spent at least 3 months in a control phase; every 3 months a cluster was randomized to enter the intervention phase. Data analysis was performed from October 15, 2023, to March 24, 2025.

**Interventions:**

Electronically generated, individualized reports that contained prioritized opportunities for deprescribing in older adults were paired with preexisting quarterly medication reviews. Deprescribing reports were accessed through a secure viewer.

**Main Outcomes and Measures:**

The primary outcome was the proportion of residents with 1 or more PIMs deprescribed in the control phase vs intervention measured every 3 months after a medication review. For the primary outcome, an adjusted odds ratio (AOR) was calculated using a generalized linear model with a logit link, controlling for the effect of the intervention and adjusted for the number of PIMs, age, sex, language, and period as fixed effects and participants nested within sites as random effects.

**Results:**

A total of 725 residents participated in the study (median [IQR] age, 84 [76-90] years; 478 [65.9%] female). The median (IQR) number of medications was 10 (7-13), and the median (IQR) number of PIMs was 3 (2-4). In the control phase, the proportion of residents with 1 or more PIMs deprescribed was 92 of 725 (12.7%) compared with 226 of 621 (36.4%) during the intervention (AOR, 1.58; 95% CI, 1.07-2.34), in favor of the intervention.

**Conclusions and Relevance:**

This study found that electronic decision support paired with the usual workflow could render the deprescribing process scalable and effective. These results suggest that medication reviews should incorporate deprescribing as part of usual care.

**Trial Registration:**

ClinicalTrials.gov Identifier: NCT04762303

## Key Points

**Question:**

Does pairing clinical decision support with routine medication reviews conducted by health care practitioners in long-term care homes increase deprescribing for older adults compared with usual care?

**Findings:**

This stepped-wedge cluster randomized trial of 725 residents in 5 long-term care homes in New Brunswick, Canada, found that pairing electronic deprescribing reports with usually occurring medication reviews resulted in a 23.7% increase in deprescribing.

**Meaning:**

Electronic deprescribing interventions integrated into the usual clinical workflow of long-term care homes can augment deprescribing, suggesting that this scalable intervention could become the standard of care for medication reviews in this setting.

## Teaser

This stepped-wedge cluster randomized trial of long-term care home residents assesses the efficacy of an evidence-based electronic decision support tool for deprescribing potentially inappropriate medications.

## Introduction

Potentially inappropriate prescribing (PIP) occurs when medications that carry a higher risk of harm than benefit are prescribed.^1,2,3^ It occurs more often among older adults in the setting of polypharmacy (taking multiple medications) and is costly and harmful.^1,4,5^ PIP and potentially inappropriate medications (PIMs) contribute to excess adverse drug events, such as falls, fractures, cognitive decline, hospitalization, and death.^6,7,8^ The direct and indirect costs are also immense for society and payers.^9^ The problem is more pronounced for older adults living in nursing homes (long-term care [LTC] homes).^10^ Depending on the screening criteria used, in some studies,^10^ the prevalence ranges from 67.8% to 87.7% of nursing home residents.

Residents of LTC homes are heavily affected by PIP for several reasons. First, the population often has complex medical conditions that require treatment with multiple medications, with some medications being beneficial and indicated. Second, many (69%) are diagnosed with a neurocognitive disorder, and as many as 50% have responsive behaviors.^11^ These conditions lead to off-label PIP of antipsychotics or sedatives.^3,12^ Other examples of common PIMs prescribed to manage complex symptoms, such as pain, anxiety, and insomnia, include antidepressants, anticholinergics, gabapentinoids, and opioids.^13^ Third, on average, in patients who have lived to an older age, some prescriptions may have accumulated over time without a proper reassessment (eg, proton pump inhibitors and multiple daily doses of calcium and vitamin D), and some medications may have been added to treat the adverse effects of other medications through prescribing cascades.^14^ Fourth, medications used for prevention may have persisted (eg, cholesterol-lowering medications, primary prevention aspirin, and cholinesterase inhibitors) despite a lack of evidence to support efficacy in this population.^15,16^ Ideally, medication regimens ought to be individualized and contextualized, factoring in a more limited life prognosis of an older adult living in a LTC home, which in some jurisdictions has been estimated at 12 to 18 months.^17^ Therefore, a shift in the goals of intervention to prioritize quality of life and a reduction in pill burden is reasonable. Taken together, these factors support a process to reduce PIP. A promising intervention is deprescribing, which is the structured process of stopping medications, reducing medications, or changing to a safer alternative, through shared decision-making, keeping in mind a person or family’s goals or health priorities.^4,5^

Deprescribing initiatives have been successful in community, acute care, and LTC environments^18,19,20,21^; in LTC, studies tend to be smaller, observational in nature, or address only 1 or 2 medication classes.^22,23,24,25^ Barriers to deprescribing in LTC are complex and include competing priorities for staff or lack of staffing (similar to acute care), a lack of reimbursement, a perception by family members that deprescribing equates to withdrawing care, and lack of training and resources.^26^ Computer decision support tools have previously been tested in LTC to improve health care delivery.^27^ We therefore sought to integrate and assess an evidence-based, Canadian-made electronic deprescribing decision support tool in LTC^20^ using a stepped-wedge cluster randomized trial to account for temporal trends and allow for all study sites to eventually receive the intervention.

## Methods

We built a web-based application, the Polypharmacy App, to provide LTC health care practitioners access to MedSafer (MedSafer Corp), an electronic decision support tool for deprescribing. MedSafer was previously effective for deprescribing in a large cluster randomized clinical trial in acute care, a small pilot in LTC, and other studies.^20,22,28^ In the current study, we tested MedSafer in a cluster randomized trial across 5 LTC homes in New Brunswick, Canada. Individual consent for the effectiveness of the primary intervention was waived by Horizon Health Network because the intervention of offering deprescribing opportunities based on existing guidelines was considered best practice; written informed consent was obtained for surveys, questionnaires, and interviews. The full study was granted ethics approval by the Horizon Health Network. The study followed the Consolidated Standards of Reporting Trials (CONSORT) reporting guideline. The trial protocol can be found in Supplement 1.

### Design, Study Population, Setting, and Randomization

**Figure 1.**
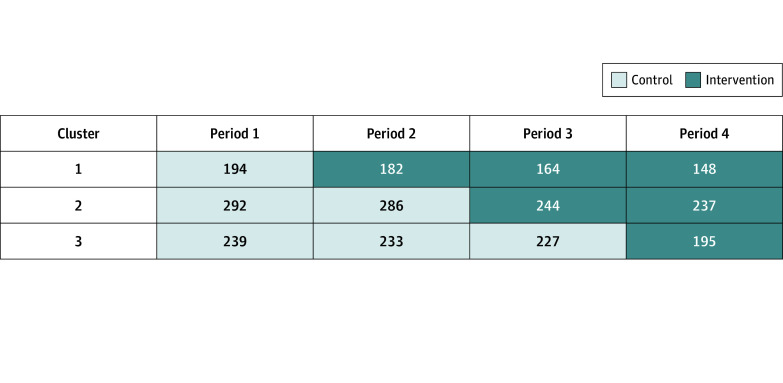
Study Clusters and Time Spent in the Control and Intervention Phases The number of residents decreases in each cluster across each period from attrition due to death (n = 143) or transfer without return (n = 2).

The study took place during 15 months from August 1, 2021, to October 31, 2022, during the COVID-19 pandemic. In Canada, the types of costs covered in nursing homes vary across the country, and homes are often a final destination (residents rarely transit through to another living arrangement). Nursing homes are a mixture of public and privately owned (which can be for profit or nonprofit).^29^ Most nursing homes in New Brunswick are private and nonprofit.^29^ Most individuals are eligible for the provincial prescription drug program and receive approved medications at no cost. Further context about the Canadian and New Brunswick LTC model is provided in the eMethods in Supplement 2. Five nursing homes were assigned to 1 of 3 clusters to roughly balance the number of residents in each cluster (the largest home was 1 cluster, and 2 smaller homes were combined to make up each of the 2 other clusters). Each cluster participated in a control phase followed by an intervention phase. The order of entry of each cluster into the intervention phase was determined centrally by a randomized sequence. Allocation was concealed until 4 weeks before the transition, which was chosen to balance the time required to prepare for the intervention with the risk of contamination. A site assessment was completed, and implementation was customized for each LTC home. A new cluster entered the intervention phase every 3 months (Figure 1). Once a cluster entered the intervention phase, practitioners could access or receive reports for the same resident in the cluster at each quarterly medication review as well as an additional report if the resident returned to the nursing home after an acute care hospitalization. Each home participated on a voluntary basis with involvement of their medical, pharmacy, and nursing leadership. Individual prescribers in the homes were encouraged to participate but were permitted to opt out of receiving the intervention. Prescribers worked at a single site. There was no financial incentive to participate.

At the start of the study, residents 65 years and older living in 1 of the 5 LTC homes and prescribed 1 or more PIMs during the study were eligible. Information on race and ethnicity was not available in the electronic health records. Residents who were newly admitted after the first 3 months were not included because we hypothesized that prescribers might hesitate to deprescribe until familiar with a new resident. Residents who died or were transferred to a hospital or to another location without returning to the home could contribute data to the study if they had at least 1 medication review in the control phase. Residents could also leave the study if they were hospitalized and were permitted to reenter the study and contribute data if they were discharged back to the same home during the study period.

### Description of the Intervention

During the control period, residents received usual care, which consisted of mandatory medication reviews every 3 months. There is no standardized procedure for medication reviews in New Brunswick nursing homes; any deprescribing that takes place depends on local and individual health care practitioner practice. Each nursing home in the study had between 2 and 5 prescribers (physicians or nurse practitioners) responsible for making medication changes after medication reviews. Each home also had access to a pharmacist, whose involvement in medication reviews varied, as is typical of Canadian nursing homes.^30^

### Software and Assessments

In New Brunswick, all nursing homes use Momentum software, version 9 (Momentum Software) to document interRAI Long-Term Care Facilities assessments. The interRAI assessments contain information about medical conditions (coded as *International Statistical Classification of Diseases and Related Health Problems, Tenth Revision [ICD-10]* or iCODES^31^; eg, iCODE C4 indicates “acute change in mental status”) and medications (coded by the Canadian Drug Identification Number) as well as sociodemographic information, and variables that describe physical function. Comorbidities and medications recorded in interRAI are updated quarterly. Assessments are completed by an interRAI assessor with input from nursing staff.

The Polypharmacy App was created as a secure way of visualizing MedSafer deprescribing opportunities in a web-based system that adhered to privacy and security principles (eAppendix 2 in Supplement 2. The intervention was compliant with the Personal Information Protection and Electronic Documents Act of Canada.^32^ The app contained data on medications and comorbidities via a linkage with Momentum software. The app assigned a unique identifier to each person, anonymized the data, isolated and transformed the relevant data elements, and then used the MedSafer application programming interface to obtain a report of deprescribing opportunities (eAppendix 1 in Supplement 2). These reports were then reidentified within the Polypharmacy App and made available to prescribers to securely visualize during medication reviews at the point of care. Reports could be viewed directly in the app or printed and placed in resident medical records (based on prescriber preference).

Deprescribing opportunities included recommendations from Choosing Wisely,^33,34,35^ the American Geriatrics Society,^3^ and the Screening Tool of Older Person’s Prescriptions (STOPP)^12^ and have been previously described.^20^ The reports prioritized opportunities for deprescribing based on expert consensus as (1) high risk (harms outweigh benefits for most), (2) intermediate risk (harms must be weighed against benefits and clinical judgment is required), or (3) drugs of little added value (examples provided in the eMethods in Supplement 2). Individualized reports also included tapering instructions when indicated and links to additional patient materials for some medication classes (eg, sedative hypnotics and proton pump inhibitors).^36,37^ A message was also displayed if no deprescribing opportunities were identified for a given resident.

### Data Collection

Medication data were compared from sequential interRAI datasets using the unique identifier attached to each dataset. PIMs identified in the baseline dataset were compared with the next dataset (3 months later) transmitted with the same unique identifier to determine whether any PIMs had been stopped (no longer listed) or whether a PIM dose had been reduced. Safety data (eg, falls, delirium, and use of restraints) were extracted quarterly from interRAI datasets.

### Outcomes

The primary outcome was the proportion of residents with 1 or more PIMs deprescribed after a medication review in the intervention vs control phases assessed quarterly (follow-up period of 3 months). Medication changes were categorized as deprescribing if the medication was stopped, tapered (gradually reduced to STOPP), or dose reduced (continued at a lower dose). Switches within the same class (eg, a switch from pantoprazole to lansoprazole) were not included. Residents were considered to have been deprescribed in the control or intervention phases if they were deprescribed at least once during the period considered. Due to repeated measurements, residents contributed data to both the control and intervention phases (eMethods in Supplement 2).

Secondary outcomes were individual medication classes deprescribed, falls, use of restraints, and delirium. Tertiary outcomes related to the usefulness and feasibility of the intervention, as well as resident and caregiver attitudes toward deprescribing (to be reported separately).

### Sample Size and Power Calculation

With 750 residents, we had 80% power to detect a 20% absolute reduction in the primary outcome with an α = .05. On the basis of the literature, the rate of deprescribing with usual care was estimated at 20%.^38^ We used an expected cluster correlation coefficient of 0.03.^38^ For a 12-month study, we planned for 3 clusters of approximately 150 to 300 residents and 4 periods.

### Statistical Analysis

Baseline characteristics were combined for the cohort because residents could contribute data to both the intervention and control groups. Baseline characteristics were expressed as numbers (percentages) for categorical variables and medians (IQRs) for continuous variables. A resident could meet the primary outcome if 1 or more PIMs were deprescribed at any time in the control phase. Similarly, a resident met the primary outcome if 1 or more PIMs were deprescribed at any time during the intervention phase. For the primary outcome, we used a generalized linear model with a logit link, evaluated for the effect of the intervention, and adjusted for the number of PIMs, age, sex, language, and period as fixed effects. We adjusted for cluster, site within cluster, and participant within site as random effects. Adjusted odds ratios (AOR) and 95% CIs were estimated from the model parameters. This analysis was restricted to data from residents who were taking at least 1 PIM during the study and who survived the first 3 months. The study was analyzed according to intention to treat. For secondary outcomes, when the number of events permitted, an identical analysis was conducted. Otherwise, unadjusted proportions were presented. Two-sided α < .05 was considered statistically significant. All analyses were conducted using SAS software, version 9.4 (SAS Institute Inc). Data analysis was performed from October 15, 2023, to March 24, 2025.

## Results

**Figure 2.**
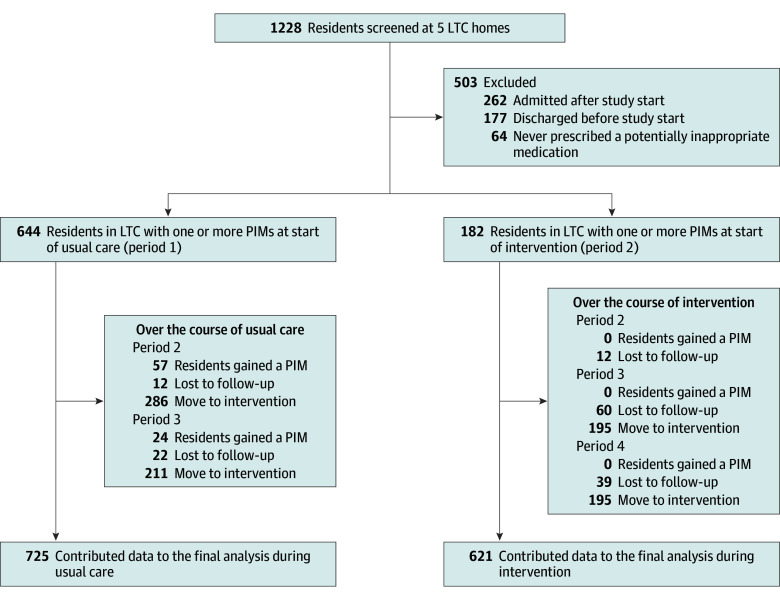
Study Flow Diagram Residents could gain or lose eligibility throughout the study based on the presence of one or more potentially inappropriate medications (PIMs) and are not mutually exclusive with residents who were lost to follow-up. Residents lost to follow-up could contribute data to usual care, the intervention phase, or both. Numbers are not additive. LTC indicates long-term care.

A total of 1228 residents were admitted to 1 of the 5 nursing homes at the start of the study; of these, 725 (59.0%) had 1 or more PIMs prescribed during the study (Figure 2). All 725 eligible residents contributed data to the control phase, and 621 (85.7%) contributed data to the intervention. The median (IQR) age was 84 (76-90) years, 478 (65.9%) were women and 247 (34.1%) were men, and 629 (86.8%) spoke English as a first language (Table 1). The prevalence of dementia was 62.7%. During the study, 143 residents (19.7%) died, and 2 were transferred to another LTC home. When surveyed, half of the prescribers accessed the app directly, and half of the prescribers requested printed reports. App access was quarterly (at the time of medication reviews). Completion rate of interRAI assessments was occasionally delayed due to the pandemic (most notable in period 2) (Table 2) but continued throughout the study.

**Table 1.** Study Patient Characteristics

Characteristic	No. (%) of patients^a^ (N = 725)
Age, median (IQR), y	84 (76-90)
Sex	
Female	478 (65.9)
Male	247 (34.1)
First language spoken or written	
English	629 (86.8)
French	93 (12.8)
No. of medications, median (IQR)^b^	10 (7-13)
No. of PIMs, median (IQR)^c^	3 (2-4)
Alzheimer disease	117 (16.1)
Dementia other than Alzheimer disease	338 (46.6)
Coronary heart disease	163 (22.5)
Hypertension	119 (16.4)
Stroke or CVA	155 (21.4)
Anxiety	230 (31.7)
Depression	295 (40.7)
Bipolar disease	16 (2.2)
Schizophrenia	35 (4.8)
Cancer	91 (12.6)
Diabetes	205 (28.3)
Gastroesophageal reflux disease, history of gastrointestinal bleed, or peptic ulcer disease	117 (16.1)

Abbreviations: CVA, cardiovascular accident; PIM, potentially inappropriate medication.

^a^ Unless otherwise indicated.

^b^ Regular and as needed.

^c^ Those with rules that were flagged by the deprescribing app.

**Table 2.** Unadjusted Proportions of Deprescribing of 1 or More Potentially Inappropriate Medications by Cluster, Site, Period, and Intervention Status

Cohort and site	Period				Total	
	1	2	3	4	Control	Intervention
**Cluster 1**						
Site 1						
No. of patients	194	180	157	148	194	182
No. (%) of PIMs deprescribed	3 (1.6)	34 (18.9)	35 (22.3)	44 (29.7)	3 (1.6)	85 (46.7)
**Cluster 2**						
Site 2						
No. of patients	152	165	111	144	165	149
No. (%) of PIMs deprescribed	1 (0.7)	25 (15.2)	15 (13.5)	49 (34.0)	26 (15.8)	62 (41.6)
Site 3						
No. of patients	109	112	28	93	127	95
No. (%) of PIMs deprescribed	0	14 (12.5)	2 (7.1)	9 (9.7)	14 (11.0)	11 (11.6)
Cluster 2 subtotal						
No. of patients	261	277	139	237	292	244
No. (%) of PIMs deprescribed	1 (0.4)	39 (14.1)	17 (12.2)	58 (24.5)	40 (13.7)	73 (29.9)
**Cluster 3**						
Site 4						
No. of patients	130	141	150	144	165	144
No. (%) of PIMs deprescribed	3 (2.3)	23 (16.3)	13 (8.7)	50 (34.7)	36 (21.8)	50 (34.7)
Site 5						
No. of patients	59	66	41	51	74	51
No. (%) of PIMs deprescribed	2 (3.4)	8 (12.1)	7 (17.1)	18 (35.3)	13 (17.6)	18 (35.3)
Cluster 3 subtotal						
No. of patients	189	207	191	195	239	195
No. (%) of PIMs deprescribed	5 (2.7)	31 (15.0)	20 (10.5)	68 (34.9)	49 (20.5)	68 (34.9)
**Control**						
No. of patients	644	484	191	0	725	0
No. (%) of PIMs deprescribed	9 (1.4)	70 (14.5)	20 (10.5)	0	92 (12.7)	0
**Intervention**						
No. of patients	NA	180	296	580	0	621
No. (%) of PIMs deprescribed	NA	34 (18.9)	52 (17.6)	170 (29.3)	0	226 (36.4)

Abbreviations: NA, not applicable; PIM, potentially inappropriate medication.

### Baseline Data

The median (IQR) number of medications was 10 (7-13), and the median (IQR) number of PIMs was 3 (2-4). The most commonly used PIMs were proton pump inhibitors (403 [55.6%]), anticoagulants (269 [37.1%]), and benzodiazepines or sedative hypnotics (283 [39.0%]).

### Outcomes

During the control phase, 92 residents (12.7%) had 1 or more PIMs deprescribed vs 226 (36.4%) residents in the intervention phase (risk difference, 23.7% [95% CI, 19.2%-28.2%]; number needed to treat of 4). This corresponded to an AOR of 1.58 (95% CI, 1.07-2.34) (eTable 1 in Supplement 2).

The most common classes of medications deprescribed during the intervention were opioids, antipsychotics, docusate, and benzodiazepines or sedative hypnotics (Table 3). Although the study was not powered for an effect on individual medication classes, medications that were least likely to be affected by the intervention were nonsteroidal anti-inflammatories (more likely to be deprescribed during the control period) and gabapentinoids (rarely deprescribed during the control or intervention period). Falls were statistically significantly higher in the intervention group than the control group (149 [20.6%] vs 7 [17.1%]; AOR, 1.77; 95% CI, 1.15-2.71) (eTable 2 in Supplement 2). Use of restraints was numerically higher in the intervention group (124 [20.0%] vs 116 [16.0%]) but after adjustment was no longer significant (AOR, 1.95; 95% CI, 0.52-7.37) (eTable 3 in Supplement 2). The prevalence of delirium was low overall, so we only report the unadjusted proportion (19 [2.6%] in the control phase and 29 [4.7%] in the intervention phase).

**Table 3.** Most Common Classes of Potentially Inappropriate Medications Deprescribed by Intervention Status

Specific PIM	No. (%) of patients			
	Control (n = 725)		Intervention (n = 621)	
	Receiving	Deprescribed	Receiving	Deprescribed
Proton pump inhibitors	403 (55.6)	6 (1.5)	362 (54.4)	29 (8.0)
Benzodiazepines and sedative hypnotics	283 (39.0)	25 (8.8)	247 (39.8)	42 (17.0)
Antiplatelet and anticoagulant combination therapy	269 (37.1)	4 (1.5)	237 (38.2)	10 (4.2)
Antipsychotics	172 (23.7)	7 (4.1)	165 (26.6)	39 (23.6)
Gabapentinoids	149 (20.6)	4 (2.7)	127 (20.5)	4 (3.1)
Opioids	126 (17.4)	16 (12.7)	113 (18.2)	31 (27.4)
Nonsteroidal anti-inflammatories	75 (10.3)	19 (25.3)	59 (9.5)	9 (15.3)
Docusate	52 (7.2)	0	54 (8.7)	10 (18.5)
Sulfonylureas	31 (4.3)	1 (3.2)	24 (3.9)	1 (4.2)
High-dose iron salts	31 (4.3)	1 (3.2)	27 (4.3)	2 (7.4)

Abbreviation: PIM, potentially inappropriate medication.

## Discussion

We conducted a large stepped-wedge randomized trial of electronic decision support for deprescribing in LTC and found that this intervention significantly increased the odds of deprescribing (AOR, 1.58) compared with usual care. A systematic review of various interventions aimed at increasing deprescribing in LTC found deprescribing interventions effective, and subgroup analyses suggested that medication review–directed deprescribing interventions could reduce all-cause mortality and falls.^39^ In our study, although the intervention was effective, there was an increase in falls in the intervention group. The association with falls may have been causal (due to less sedation or patients moving more) or may have been related to changes in LTC staffing, reduced access to family members, and increased use of antipsychotics^40^ observed in 2020 and 2021 in Canada during the COVID-19 pandemic (an increased use of restraints was similarly observed during this time).^40^ The increase in falls was less likely due to failure to appropriately taper medications because tapering instructions were provided, although we did not monitor for adherence to tapering by prescribers.

To date, deprescribing has yet to be scaled up and sustained in the LTC environment due to a number of known complex barriers.^24,26^ Insufficient time and human resources, lack of coordination between health care settings and practitioners, and negative social influences (eg, a lack of understanding of the benefits of deprescribing and a lack of stakeholder buy-in) are often cited as barriers.^26,41,42^ To overcome insufficient resources, a prior study successfully leveraged health information technology to identify residents of LTC who might benefit from medication monitoring plans and demonstrated reduced rates of delirium, hospitalization, and death (N = 491).^43^ Our electronic decision support tool was designed to identify residents with 1 or more PIMs, reduce the cognitive load required for deprescribing, and provide a nudge to the clinician; the result was increased deprescribing.

This is in contrast with some prior electronic deprescribing interventions that have shown poor uptake and failure to show an impact of deprescribing (eg, the Software Engine for the Assessment and Optimisation of Drug and Non-drug Therapy in Older Persons [SENATOR] trial).^44^ In part, this may have been due to the timing of reports and a lower proportion of clinically significant recommendations. In contrast, when we embedded an electronic deprescribing intervention with usual processes and paired reports with medication reviews in acute care, we increased deprescribing.^20^ An important lesson learned from MedSafer studies has been to pair the intervention with the usual workflow, so we worked with study sites to determine how the app could best integrate into their medication review process, which may have increased uptake.

In LTC homes, medication reviews are mandatory in many jurisdictions. However, without structure or oversight, they often lead to medications simply being represcribed. With paired decision support, the cognitive burden to cross-reference medications, medical conditions, and guidelines is reduced. Some pharmacists and prescribers are responsible for hundreds of residents; in such scenarios, use of technology and the principle of a quality-improvement nudge can be extremely effective. By creating a report with all the information in one place, along with the how-to of deprescribing, and by pairing the report with usual workflow, the issue of alert fatigue is better addressed. Real-time decision support at the time of new prescriptions could become irritating to the prescriber and risk opportunities being silenced or ignored due to alert fatigue. Instead, our intervention nudged prescribers to regularly reassess PIMs, which is important as people age and goals of care shift.

### Limitations

This study had limitations worth discussing. First, our study took place during the COVID-19 pandemic, which may have influenced how homes completed interRAI assessments. In some periods and in some homes, assessments were delayed. Still, assessments continued; to be included in the study, participants had to have at least 1 assessment, and most had 2 to 4. The pandemic may also have increased the number of PIMs, although numbers appeared similar to those in prepandemic studies.^22^ Furthermore, because the data were entered by LTC home staff, there may have been data entry errors; we were not able to validate the information because we were unable to go into homes to audit data entry. Second, our study only had 3 clusters. As such, it is possible that biases related to temporal trends were incompletely accounted for. Third, we did not power this study for a reduction in adverse drug events or for individual medication classes. Based on prior research,^20^ this would require a study of 8000 or more participants, a substantially larger budget, and a means of validating the events. Fourth, we did not examine the long-term persistence of deprescribing, and the follow-up period was short (although this may be less relevant in the LTC setting, where life expectancy is restricted). It is possible that medications that were deprescribed were restarted later. Fifth, we found an unexpected significant increase in falls (and numerically increased cases of delirium and use of restraints), which may have related to the intervention itself, but as discussed may also have related to the timing of the intervention during the COVID-19 pandemic. Many countries were faced with reduced staffing in LTC to manage increased movement and activity brought on from deprescribing sedating medications and perhaps less equipped to monitor for emerging symptoms during tapering. Use of restraints independently increased in LTC during the pandemic across the country, and rates were highest in the province of study (23.9%); however, the overall rate of restraint use was lower in the study homes (16%-20%).^40^ In addition, because the same patients were in both the control and intervention groups, they aged throughout the study. Although we adjusted for age, we did not adjust for an additional 3 to 9 months in the LTC environment, which might have negatively impacted these safety outcomes.

## Conclusions

Deprescribing interventions can be effective in LTC homes, and use of electronic decision support paired within the usual workflow is a potentially scalable candidate intervention. Although most deprescribing interventions are resource intensive, this was a low-touch intervention that made use of data routinely collected in the electronic interRAI assessment. The next steps would be to scale up the intervention at the level of a larger health organization to demonstrate longevity of the intervention and impact on adverse drug events. While trials are performed, PIP in LTC is common enough that steps can be taken to rationalize medications during routinely performed medication reviews. Incorporating deprescribing should be encouraged as best practice where time and resources permit. Given a finding of increased falls in our study, ensuring medications are appropriately tapered and that homes are well resourced during a deprescribing intervention is important for safety. Whether we need further proof of impact on outcomes other than PIP is debatable; arguably, residents, families, and the health care system should hot have to bear the burden of medications that are no longer needed or potentially harmful.

## Notes

Supplement 1.
Trial Protocol

Supplement 2. **eAppendix 1.** Examples of Deprescribing Opportunities Generated by MedSafer With App Screenshots **eMethods.** Supplemental eMethods **eTable 1**. Adjusted Odds of Deprescribing One or More Potentially Inappropriate Medications **eTable 2.** Adjusted Odds of Restraints Use **eTable 3.** Adjusted Odds of Having a Fall **eAppendix 2.** Description of the Deprescribing App Data Privacy and Security

Supplement 3.
Data Sharing Statement

## References

[1] Hire AJ, Franklin BD. Potentially inappropriate prescribing (PIP) in older people and its association with socioeconomic deprivation-a systematic review and narrative synthesis. BMC Geriatr. 2024;24(1):651. doi:10.1186/s12877-024-04858-w 39095729 PMC11295679

[2] Guillot J, Maumus-Robert S, Marceron A, Noize P, Pariente A, Bezin J. The burden of potentially inappropriate medications in chronic polypharmacy. J Clin Med. 2020;9(11):3728. doi:10.3390/jcm9113728 33233595 PMC7699788

[3] 2023 American Geriatrics Society Beers Criteria® Update Expert Panel. American Geriatrics Society 2023 updated AGS Beers Criteria® for potentially inappropriate medication use in older adults. J Am Geriatr Soc. 2023;71(7):2052-2081. doi:10.1111/jgs.18372 37139824 PMC12478568

[4] Reeve E, Gnjidic D, Long J, Hilmer S. A systematic review of the emerging definition of ‘deprescribing’ with network analysis: implications for future research and clinical practice. Br J Clin Pharmacol. 2015;80(6):1254-1268. doi:10.1111/bcp.12732 27006985 PMC4693477

[5] Scott IA, Hilmer SN, Reeve E, . Reducing inappropriate polypharmacy: the process of deprescribing. JAMA Intern Med. 2015;175(5):827-834. doi:10.1001/jamainternmed.2015.0324 25798731

[6] Lavan AH, Gallagher P. Predicting risk of adverse drug reactions in older adults. Ther Adv Drug Saf. 2016;7(1):11-22. doi:10.1177/2042098615615472 26834959 PMC4716390

[7] Gomes D, Herdeiro MT, Ribeiro-Vaz I, Ferreira PL, Roque F. Adverse drug reactions and potentially inappropriate medication in older patients: analysis of the Portuguese pharmacovigilance database. J Clin Med. 2022;11(8):2229. doi:10.3390/jcm11082229 35456322 PMC9029593

[8] Ross SB, Wu PE, Atique A, . Adverse drug events in older adults: review of adjudication methods in deprescribing studies. J Am Geriatr Soc. 2020;68(7):1594-1602. doi:10.1111/jgs.16382 32142161

[9] Huon JF, Sanyal C, Gagnon CL, . The cost of potentially inappropriate medications for older adults in Canada: a comparative cross-sectional study. J Am Geriatr Soc. 2024;72(11):3530-3540. doi:10.1111/jgs.19164 39235969

[10] Díaz Planelles I, Navarro-Tapia E, García-Algar Ó, Andreu-Fernández V. Prevalence of potentially inappropriate prescriptions according to the new STOPP/START criteria in nursing homes: a systematic review. Healthcare (Basel). 2023;11(3):422. doi:10.3390/healthcare11030422 36766997 PMC9914658

[11] Canadian Institute for Health Information. Dementia in long-term care. Published online 2024. Accessed October 20, 2024. https://www.cihi.ca/en/dementia-in-canada/dementia-care-across-the-health-system/dementia-in-long-term-care

[12] O’Mahony D, Cherubini A, Guiteras AR, . STOPP/START criteria for potentially inappropriate prescribing in older people: version 3. Eur Geriatr Med. 2023;14(4):625-632. doi:10.1007/s41999-023-00777-y 37256475 PMC10447584

[13] Jester DJ, Molinari V, Zgibor JC, Volicer L. Prevalence of psychotropic polypharmacy in nursing home residents with dementia: a meta-analysis. Int Psychogeriatr. 2021;33(10):1083-1098. doi:10.1017/S1041610220004032 33407955

[14] McCarthy LM, Savage R, Dalton K, . ThinkCascades: a tool for identifying clinically important prescribing cascades affecting older people. Drugs Aging. 2022;39(10):829-840. doi:10.1007/s40266-022-00964-9 36107399 PMC9477172

[15] Deprescribing.org. Deprescribing guidelines and algorithms. Accessed September 9, 2022. https://deprescribing.org/resources/deprescribing-guidelines-algorithms/

[16] Mack DS, Baek J, Tjia J, Lapane KL. Statin discontinuation and life-limiting illness in non-skilled stay nursing homes at admission. J Am Geriatr Soc. 2020;68(12):2787-2796. doi:10.1111/jgs.16777 33270223 PMC8127623

[17] Hoben M, Chamberlain SA, Gruneir A, . Nursing home length of stay in 3 Canadian health regions: temporal trends, jurisdictional differences, and associated factors. J Am Med Dir Assoc. 2019;20(9):1121-1128. doi:10.1016/j.jamda.2019.01.144 30879948

[18] Omuya H, Nickel C, Wilson P, Chewning B. A systematic review of randomised-controlled trials on deprescribing outcomes in older adults with polypharmacy. Int J Pharm Pract. 2023;31(4):349-368. doi:10.1093/ijpp/riad025 37155330

[19] Thillainadesan J, Gnjidic D, Green S, Hilmer SN. Impact of deprescribing interventions in older hospitalised patients on prescribing and clinical outcomes: a systematic review of randomised trials. Drugs Aging. 2018;35(4):303-319. doi:10.1007/s40266-018-0536-4 29541966

[20] McDonald EG, Wu PE, Rashidi B, . The MedSafer study-electronic decision support for deprescribing in hospitalized older adults: a cluster randomized clinical trial. JAMA Intern Med. 2022;182(3):265-273. doi:10.1001/jamainternmed.2021.7429 35040926 PMC8767487

[21] Lee TC, Bortolussi-Courval É, McCarthy LM, McDonald EG. Deprescribing is associated with reduced readmission to hospital: an updated meta-analysis of randomized controlled trials. J Am Geriatr Soc. 2025;73(1):302-305. doi:10.1111/jgs.19166 39238319

[22] Perri GA, Bortolussi-Courval É, Brinton CD, . MedSafer to support deprescribing for residents of long-term care: a mixed-methods study. Can Geriatr J. 2022;25(2):175-182. doi:10.5770/cgj.25.545 35747414 PMC9156423

[23] Cateau D, Ballabeni P, Niquille A. Effects of an interprofessional deprescribing intervention in Swiss nursing homes: the Individual Deprescribing Intervention (IDeI) randomised controlled trial. BMC Geriatr. 2021;21(1):655. doi:10.1186/s12877-021-02465-7 34798826 PMC8603597

[24] Balsom C, Pittman N, King R, Kelly D. Impact of a pharmacist-administered deprescribing intervention on nursing home residents: a randomized controlled trial. Int J Clin Pharm. 2020;42(4):1153-1167. doi:10.1007/s11096-020-01073-6 32494991

[25] Kua CH, Yeo CYY, Tan PC, . Association of deprescribing with reduction in mortality and hospitalization: a pragmatic stepped-wedge cluster-randomized controlled trial. J Am Med Dir Assoc. 2021;22(1):82-89.e3. doi:10.1016/j.jamda.2020.03.012 32423694

[26] Heinrich CH, Hurley E, McCarthy S, McHugh S, Donovan MD. Barriers and enablers to deprescribing in long-term care facilities: a ‘best-fit’ framework synthesis of the qualitative evidence. Age Ageing. 2022;51(1):afab250. doi:10.1093/ageing/afab250 35077555

[27] Abdellatif A, Bouaud J, Lafuente-Lafuente C, Belmin J, Séroussi B. Computerized decision support systems for nursing homes: a scoping review. J Am Med Dir Assoc. 2021;22(5):984-994. doi:10.1016/j.jamda.2021.01.080 33639117

[28] Bortolussi-Courval É, Podymow T, Trinh E, . Electronic decision support for deprescribing in patients on hemodialysis: clinical research protocol for a prospective, controlled, quality improvement study. Can J Kidney Health Dis. Published online June 26, 2023. doi:10.1177/20543581231165712 37435299 PMC10331104

[29] Canadian Institute for Health Information. Long-term care homes in Canada: how many and who owns them? Accessed March 22, 2025. https://www.cihi.ca/en/long-term-care-homes-in-canada-how-many-and-who-owns-them

[30] Lee SWH, Mak VSL, Tang YW. Pharmacist services in nursing homes: a systematic review and meta-analysis. Br J Clin Pharmacol. 2019;85(12):2668-2688. doi:10.1111/bcp.14101 31465121 PMC6955407

[31] Canadian Institute for Health Information. CIHI coding reference guide for the interRAI Home Care. Accessed April 20, 2025. https://secure.cihi.ca/free_products/cihi-coding-reference-guide-interrai-hc-manual-2025-en.pdf

[32] Office of the Privacy Commission of Canada. The Personal Information Protection and Electronic Documents Act (PIPEDA). December 8, 2021. Accessed October 20, 2024. https://www.priv.gc.ca/en/privacy-topics/privacy-laws-in-canada/the-personal-information-protection-and-electronic-documents-act-pipeda/

[33] Choosing Wisely Canada. Gastroenterology. Accessed September 9, 2022. https://choosingwiselycanada.org/recommendation/gastroenterology/

[34] Choosing Wisely Canada. Psychiatry. Accessed September 9, 2022. https://choosingwiselycanada.org/recommendation/psychiatry/

[35] Choosing Wisely Canada. Less sedatives for your older relatives. Accessed March 2, 2024. https://choosingwiselycanada.org/toolkit/less-sedatives-for-your-older-relatives/

[36] Tannenbaum C, Martin P, Tamblyn R, Benedetti A, Ahmed S. Reduction of inappropriate benzodiazepine prescriptions among older adults through direct patient education: the EMPOWER cluster randomized trial. JAMA Intern Med. 2014;174(6):890-898. doi:10.1001/jamainternmed.2014.949 24733354

[37] Deprescribing Network. Do I still need this medication? is deprescribing for you? Accessed October 20, 2024. https://www.deprescribingnetwork.ca/useful-resources

[38] Nadeau M-E, Henry JL, Lee TD, . Spread and scale of an electronic deprescribing software to improve health outcomes of older adults living in nursing homes: study protocol for a stepped wedge cluster randomized trial. Trials. 2021;22(1):763. doi:10.1186/s13063-021-05729-0 34727956 PMC8561344

[39] Kua CH, Mak VSL, Huey Lee SW. Health outcomes of deprescribing interventions among older residents in nursing homes: a systematic review and meta-analysis. J Am Med Dir Assoc. 2019;20(3):362-372.e11. doi:10.1016/j.jamda.2018.10.026 30581126

[40] Appropriate Use Coalition. Rising rates: antipsychotic use in Canada’s LTC homes. 2024. Accessed December 6, 2024. https://ltcmeds.ca/

[41] Rowe S, Pittman N, Balsom C, Druken R, Kelly DV. Beliefs and attitudes of residents, family members and healthcare professionals regarding deprescribing in long-term care: a qualitative study. Int J Clin Pharm. 2022;44(6):1370-1379. doi:10.1007/s11096-022-01419-2 36201111

[42] Palagyi A, Keay L, Harper J, Potter J, Lindley RI. Barricades and brickwalls–a qualitative study exploring perceptions of medication use and deprescribing in long-term care. BMC Geriatr. 2016;16:15. doi:10.1186/s12877-016-0181-x 26767619 PMC4714480

[43] Lapane KL, Hughes CM, Daiello LA, Cameron KA, Feinberg J. Effect of a pharmacist-led multicomponent intervention focusing on the medication monitoring phase to prevent potential adverse drug events in nursing homes. J Am Geriatr Soc. 2011;59(7):1238-1245. 21649623 10.1111/j.1532-5415.2011.03418.x PMC3157676

[44] O’Mahony D, Gudmundsson A, Soiza RL, et al. Prevention of adverse drug reactions in hospitalized older patients with multi-morbidity and polypharmacy: the SENATOR* randomized controlled clinical trial. Age Ageing. 2020;49(4):605-614. doi:10.1093/ageing/afaa072 32484850

