# Supplementary material for: Electronic Decision Support for Deprescribing in Older Adults Living in Long-Term Care: A Stepped-Wedge Cluster Randomized Trial
Source: JAMA Netw Open. 2025 May 30;8(5):e2512931. doi: 10.1001/jamanetworkopen.2025.12931 (PMC12125643; doi:10.1001/jamanetworkopen.2025.12931)
Supplement: Supplement 1. — Trial Protocol [file jamanetwopen-e2512931-s001.pdf]

Electronic Decision Support for Deprescribing in Older Adults Living in Long Term Care

Principal Investigators:

Emily G. McDonald MD MSc[1,2], Justine L. Estey MSc[3], Cody Davenport MSc[3], Émilie Bortolussi-Courval RN[2], Jeffrey Gaudet MSc[4,5], Pierre Philippe Wilson Registe MSc MPH[6,7], Todd C. Lee MD MPH[2,8], Carole Goodine PharmD[9]

[1]Division of General Internal Medicine, Department of Medicine, McGill University Health Centre, Montreal, Quebec, Canada

[2]Division of Experimental Medicine, Department of Medicine, McGill University, Montreal, Quebec, Canada

[3]The Centre for Innovation and Research in Aging, Fredericton, New Brunswick, Canada

[4]Vitalité Health Network, New Brunswick, Canada

[5]Maritime SPOR SUPPORT Unit, Canada

[6]Université de Sherbrooke, Montréal, Québec, Canada

[7]Centre de Formation Médicale du Nouveau Brunswick, New Brunswick, Canada

[8]Division of Infectious Diseases, Department of Medicine, McGill University Health Centre, Montreal, Quebec, Canada

[9]Horizon Health Network, Pharmacy Services, Doctor Everett Chalmers Regional Hospital, Fredericton, New Brunswick, Canada

Abstract

Medication overload is an epidemic causing widespread harm, particularly to older Canadians. While most prescriptions are intended to help people live longer and healthier lives, taking multiple medications can increase frailty, and lead to dangerous side effects. As people age, the way medicines work may change and certain medicines are more likely cause problems with memory, thinking and balance. To minimize these risks, it is important to regularly review all medicines an older adult is taking. A medication review can be complicated, and it takes time. MedSafer is a computer program that helps physicians and pharmacists identify medicines that may no longer be needed or are dangerous. The software runs an analysis of a person's medications and medical conditions and produces a report with suggestions for how to simplify combinations of medications and make them safer. The process of safely reducing medications is called deprescribing. We built a computer application powered by MedSafer for nursing homes. Our Polypharmacy App was piloted at a large urban nursing home. Initial impression of the system was positive. Improvements were made and it was rebranded as MedReviewRx. We would like to test MedReviewRx in other nursing homes in New Brunswick

Methods

This will be a mixed-methods study to determine the impact of MedReviewRx in New Brunswick Nursing Homes (NBNHs). We will measure the prevalence of potentially inappropriate medications (PIMs) in NBNHs before MedReviewRx and calculate the change in PIM use after implementation. User experience with MedSafer reports and MedReviewRx will be explored. Patient and Family attitudes towards deprescribing will also be studied.

Benefits

MedReviewRx allows health care providers (prescribers, pharmacists and nurses) in nursing homes to access MedSafer analysis on a tablet or laptop. We expect that access to this information will promote deprescribing in nursing homes, reduce PIMs and result in cost savings.

Spread and scale of a Polypharmacy App to Improve Health Outcomes of Older Adults living in New Brunswick Nursing Homes

Background

Medication overload, or problematic polypharmacy, is an overwhelming problem for older adults. Nearly two thirds of community dwelling adults are taking five or more medications (CIHI, 2018) and while most medications are prescribed to help people live longer, healthier lives, the more complex a medication regimen is, the more dangerous it becomes. With each added drug, the risk of an adverse drug event (ADE) increases. A growing body of evidence suggesting that patients and physicians may not recognise ADEs and additional new medications may be prescribed to treat an existing ADE (Brath et al, 2018). This prescribing cascade further increases polypharmacy and the risk of ADEs. Aging itself is another risk factor for ADEs. As we age, changes in organ function, body weight, fat distribution, cognition, balance, function and drug receptor sensitivity can alter the way medication works. Geriatric experts from around the world have published guidelines and lists of medications which should be avoided or used cautiously in older adults. These medications are often referred to as potentially inappropriate medications or PIMs. PIMs are medications associated with a high risk of ADEs when administered to older adults, and/or of uncertain or no clinical benefit. Continued use of PIMs in later life, further increases the risk of ADEs associated with polypharmacy in older adults. Older adults living in nursing homes are at a particularly high risk as they are frail and often take ten or more medications (CIHI, 2018). Some of these medications are potentially inappropriate and increase the risk of falls, fractures, kidney injury, bleeding, and even death. The cost of polypharmacy is very high and stems from emergency department visits, hospital admissions, and direct and indirect drug costs.

Strategies to address polypharmacy are needed. One approach is the process of deprescribing, whereby a prescriber or pharmacist establishes the full list of medications a patient is taking and contextualizes them for the individual patient. Based on the medical history, a measure of frailty, and the patient and/or caregiver's values or preferences, safer classes of medications are selected, doses may be decreased, and medication may be discontinued. This process can be thought of as a "prescription check-up". Manually reviewing all drugs and cross referencing them with patient conditions and lists of inappropriate drugs in older adults requires an expert command of the literature and requires a significant amount of time. While nursing home medication reviews are mandatory in New Brunswick and most other provinces, oftentimes medications are re-prescribed without documentation of a clear rationale or assessment. Studies in acute care hospitals have demonstrated that electronic decision support can facilitate the process of deprescribing and augment the proportion of patients with one or more medications stopped upon discharge from the hospital (McDonald et al, 2019). More studies looking at electronic tools for deprescribing in nursing homes are needed to determine if an investment on the part of the government into a software that supports deprescribing is warranted.

We know that most patients and their family members are eager to stop medications, especially if they are harmful or unnecessary, and if their doctor supports discontinuing the medication (Sirois et al, 2017). Studies that have examined barriers to deprescribing have identified prescriber beliefs, attitudes, knowledge, skills, patient beliefs, health system and work setting as challenges (Anderson et al, 2014). Lack of clinician time and resources to stop medications safely and effectively is a common theme. A strategy that brings the expert knowledge of deprescribing to most prescribers and cuts down on the time it takes to perform a prescription check-up would address several of the barriers to deprescribing.

An electronic decision support tool for deprescribing called MedSafer has been studied extensively in the acute care setting (McDonald et al, 2019). In a pilot study MedSafer helped to augment the proportion of patients who were discharged from the hospital with one or more PIMs reduced or stopped. This was compared to usual care whereby a pharmacist performed a reconciliation of the home medications and ensured the list was accurate and reproduced at transitions of care. MedSafer has been studied on over 6000 hospitalized Canadians to date and appears to effectively facilitate the process of a prescription check-up by providing expert reports containing automatically generated deprescribing opportunities based on a list of medication the patient was taking at home (Best Possible Medication History) and their past medical conditions (McDonald et al, 2019).

New Brunswick (NB) has the highest proportion of older adults compared to other provinces in Canada. In NB, 20.8% of the population is over 65 years of age and this is expected to increase to 31.3% by the year 2038. Currently, about 5,570 seniors in NB live in a nursing home. Canadian Institute for Health Information (CIHI) 2016 statistics indicate that more than 60% of older adults living in nursing homes were taking 10 or more medications (CIHI,2018). This means that over 3,000 of the seniors living in New Brunswick nursing homes (NBNHs) are receiving more than 10 regularly scheduled medications. Strategies to promote regular prescription check-up are needed to reduce polypharmacy. Nursing homes have multidisciplinary teams that provide 24-hour care therefore they are an ideal environment to test new and innovative methods to promote deprescribing and reduce PIMs.

One method of monitoring medication use in nursing homes is to use interRAI assessment data. InterRAI Long-Term Care Facilities (LTCF) assessments are conducted quarterly for every person living in a NBNH. These assessments contain sociodemographic information, medical diagnoses,

information on medications used by the resident, and variables which describe cognitive and physical function. (Morris, 2011) In NB, the interRAI LTCF assessment form is completed electronically using Momentum Healthware (Momentum) software. This computer database represents a unique opportunity to automate MedSafer data entry and provide health care providers in nursing homes with a tool to identify PIMs which should be reassessed.

Funding was received from the Centre for Ageing and Brain Health Innovation to develop MedSafer for use in Canadian long-term care facilities. MedSafer researchers partnered with the Centre for Innovation and Research in Aging (CIRA) at York Care Centre (YCC) to develop a web-based application (Polypharmacy App) that allowed prescribers to visualize MedSafer reports on a desktop computer or tablet. Using an application programming interface (API), patient data derived from Momentum electronic interRAI LTCF software was anonymized and transmitted to MedSafer for analysis. MedSafer cross-references medical conditions, ICD-10 diagnosis codes and medication drug identification numbers (DINs) to generate a deprescribing opportunities report that can be used by the prescriber as a roadmap for a comprehensive prescription check-up. MedSafer report output was designed to be printed or visualized in the Polypharmacy App. Our Polypharmacy App was piloted at YCC, a large urban nursing home in Fredericton, New Brunswick. Initial impression of the system was positive. Improvements were made and the Polypharmacy App was rebranded as MedReviewRx. We would like to test MedReviewRx in other NBNHs.

The Present Study

Purpose

The purpose of the present project is to imbed deprescribing into NBNHs using MedReviewRx, and to evaluate user experience with the system.

Hypothesis: Implementing MedReviewRx in NBNHs will promote deprescribing which will lead to a decrease in the number of PIMs and a decrease in medication costs for older adults living in NBNHs.

Research questions:

1) What is the impact of MedReviewRx on the prevalence of PIMs in NBNHs?
2) Is the MedReviewRx system developed for YCC, easy to use, and does it provide useful information for health care providers in other NBNHs?
3) What are the challenges to implementing MedReviewRx in other NBNHs and what improvements are required for its widespread use?
4) What attitudes do patients and family members of patients who live in a study nursing home have towards deprescribing medication?

Methods

Participants

A subset of nursing homes in New Brunswick have agreed to participate in the study. Study participants will include older adults who live in these nursing homes and for surveys: health care providers and nursing home staff at participating nursing homes, as well as family members or substitute decision makers of older adult residents.

Study nursing homes may include:

- Shannex – Embassy Hall - Quispamsis – 72 beds

- Shannex – Monarch Hall – Riverview – 72 beds
- Loch Lomond Villa (LLV) – Saint John – 190 beds
- Spencer Nursing Home – Moncton – 200 beds
- York Care Centre (YCC) – Fredericton – 218 beds

This represents 752 nursing home beds and will provide a sample of NBNH residents and health care providers in three urban centers representing both large and mid-size long term care facilities who conduct business in English. Faubourd du Mascaret, a new bilingual 60 bed nursing home in Moncton, NB is also considering study participation.

Prescribers (physicians and nurse practitioners) and pharmacists providing care at the study nursing homes will be eligible to use MedReviewRx to conduct prescription check-ups for older adults under their care. Nursing home staff (unit clerks, administrators, interRAI co-ordinators and nurses) at the study site will be eligible to register to load LTCF assessment information into MedReviewRx and print MedSafer reports from the system for prescribers who do not use MedReviewRx. A designated contact person will be appointed by the Director of Nursing or designate at each nursing home. The study site contact will act as a liaison with the research study team and coordinate study site activities. Study site contacts will also complete a site assessment questionnaire at the beginning of the study and an interview within 3 months of initial MedReviewRx implementation. Prescribers, pharmacists, nurses and staff at study nursing homes will be recruited to complete surveys to evaluate the MedReviewRx system and MedSafer report information. At the end of the study site contacts, nursing home staff, prescribers, pharmacists and nurses will be recruited to participate in an interview to evaluate the program. Consent will be obtained for MedeviewRx users, interview and survey participants.

Adults age 65 years or older who live at a participating nursing homes and have had a quarterly interRAI LTCF assessment completed are eligible for MedSafer analysis. A waiver of patient consent is requested to load data into MedReviewRx. InterRAI LTCF assessments are completed on admission to a NBNH, and every 3 months thereafter as a standard of care. Assessment data is collected by NBNHs electronically using Momentum software. Momentum has written a customized data extract report for this project. This customized report is downloaded from Momentum and uploaded into MedReviewRx at the study site. Only the patient demographics, medical conditions, medication information and safety monitoring data required for this study are loaded into MedReviewRx. MedReviewRx removes identifiable patient information and assigns a unique identifier to each person's data set, anonymized data sets are encrypted and securely transmitted to MedSafer for analysis. The ability to download secondary interRAI assessment data on medical conditions, medication lists and safety data for each nursing home resident is essential to this research study. Without this information it will not be possible to calculate the proportion of PIMs used at the study sites, evaluate the impact of MedReviewRx on deprescribing at each site, or assess study safety endpoints. A waiver of consent is unlikely to adversely affect the welfare of individuals to whom the information relates as the information is already collected as part of usual care. Study researchers have conducted an analysis of the MedReviewRx system and appropriate measures have been taken to protect the privacy of individuals and to safeguard personal health information. It is impracticable to seek consent from all individuals to whom the secondary information relates as new nursing home residents are admitted regularly when previous residents are discharged or deceased, interRAI data is downloaded in batches by assessment date, and study endpoints require information on all residents in each study site. In addition to safeguarding information, the research team will enter into data sharing agreements with each study site to obtain permission for secondary use of information for this research study.

Adults age 65 years or older who live at a participating nursing home during the study, and their family members or substitute decision makers will be recruited to participate in a survey to explore their attitudes on medication use and deprescribing. Posters will be placed in study nursing homes; the study will be promoted verbally at resident and family council meetings and a recruitment communication will be sent by e-mail or mail to family members identified as contacts and/or substitute decision maker in the resident's admission documents. Consent will be obtained for survey completion.

Materials

This study requires:

1) Electronic documentation of medications and medical conditions in Momentum interRAI LTCF assessment software
2) MedSafer software system for identifying PIMs.
3) A secure Cloud Service to store information. This will be obtained through a subscription from Canadian Web Hosting. Canadian Web Hosting is a leader in web and cloud hosting in Canada. They specialize in hosting business and enterprise-class clients from around the world and are one of a few SSAE 16 certified service providers in Canada. They hold a heavy focus on compliance and business processes including Canadian privacy laws (PIPEDA). Canadian Web Hosting delivers a secure and scalable service. This host provider was chosen in collaboration with Missing Links Technology (MLT) based on a review of available solutions.

4) MedReviewRx, system which extracts, de-identifies and transmit data between study nursing homes and MedSafer and provide an interface for viewing MedSafer outputs on mobile devices and computers.
5) Nursing Home Assessment Questionnaire
6) Implementation Toolkit
7) User Feedback Survey
8) Acceptability and Feasibility Survey
9) Revised Patient Attitude Towards Deprescribing (rPATD) questionnaire "older adult" version for the patients and a "caregiver" version for family members and substitute decision makers.
10) Recruitment materials

Procedure

Study design

This is a hybrid type 2 effectiveness-implementation design for quality improvement research that will make use of mixed methods of evaluation. Implementation consists of using MedReviewRx. Effectiveness analysis consists of measuring the impact of the MedReviewRx on PIMs. Exploratory surveys (the mixed methods component of the study) which look at qualitative and quantitative user feedback and patient and caregiver attitudes about deprescribing will be used to explain study findings. Informal feedback, surveys and semi-structured interviews will be used to examine user experience with MedReviewRx. The Revised Patient Attitude Towards Deprescribing (rPATD) questionnaire "older adult" version for the patients and a "caregiver" version for family members/substitute decision makers will be used to describe patient and family member attitudes about deprescribing medications.

Estimated study duration is 18 months. Deployment will approximate a Stepped Wedge Cluster Randomized Trial Design. This type of study design allows us to approximate a randomized clinical trial. It has many advantages and is considered the most robust type of study design for a pragmatic quality improvement intervention. All clusters act as an internal control (before and after) as well as a control for the other clusters (external control). This novel study design addresses issues with seasonality and allows all clusters to participate in the intervention, which is an advantage when the intervention is related to quality improvement.

The study will have three clusters and each cluster having similar numbers of nursing home residents. A random generator was used to place participating nursing homes into study clusters.

Expected study clusters are:

- Cluster A – YCC 218 beds
- Cluster B – LLV 200 + Monarch 72
- Cluster C – Spencer 190 + Embassy 72

Nursing homes may experience 20 to 30% patient turnover each year due to death therefore the total number of nursing home residents who have one or more interRAI LTCF assessments during the study period could be as high as 1000. We expect that 60% of the nursing home residents will be taking more than 10 medications and at least 30% will be taking a PIM.

Each study site will appoint a site contact to load information into MedReviewRx and to communicate with the study team. Communication between study sites and researchers will occur by telephone or by Zoom using the CIRA institutional subscription when possible. The nursing home site contact or designate(s) will be e-mailed a Nursing Home Site Assessment Questionnaire for completion prior to project implementation. The purpose of this questionnaire is to determine information about the nursing

home including workflow, structure of medication reviews, physician, pharmacy and nursing support. This information will be used to inform implementation and determine where MedReviewRx fits into workflow. Study sites will also receive an implementation toolkit containing a user manual, instructions on how to use the Momentum extract report, a research study PowerPoint presentation, template for Staff/Crew Meeting “huddles”, sample announcement of study frequently asked questions and a list of deprescribing resource websites. The CIRA research manager or designate will review the Nursing Home Site Assessment Questionnaire and Toolkit resources with the study site contact during a planning meeting which will be conducted by telephone, Zoom or if permitted in person. If a nursing home site is not completing section N1 (List of all medications) of the InterRAI LTCF assessment, a research assistant will be trained to enter this information for the study site. If a research assistant is required to enter medication information into section N, the research team will work with the study site to set up an off-site location for data entry. Offsite data entry by research assistants will be used to minimize disruptions in study data entry which could occur if COVID visitor restrictions are imposed at a study site. Remote VPN access to the sites Momentum LTCF software will be provided to the research assistant(s), and arrangements will be made for transporting medication information to the research assistant. If the study nursing home uses paper Medication Administration Records (MARs), a photocopy of MARs from the study site will be sent by a bonded, traceable courier to the research assistant for offsite data entry. If the nursing home uses electronic MARs a copy of the electronic MAR will be downloaded onto an encrypted data stick and sent by bonded, traceable courier to the research assistant for offsite data entry. The frequency of data transfer will be coordinated with the nursing home site contact to align with the interRAI assessment schedule for that site.

The study will begin with a minimum three-month control phase. During the control phase, MedReviewRx will not be accessible to health care professionals at the nursing homes. This serves to

obtain baseline deprescribing levels for each nursing home. Every three to four months thereafter, a cluster of nursing homes will enter intervention mode.

<table>
<tr><th></th><th>BASELINE</th><th>1ST CYCLE</th><th>2ND CYCLE</th><th>3RD CYCLE</th></tr>
<tr><td>Cluster 1</td><td>3 months CONTROL</td><td colspan="3">9 months INTERVENTION</td></tr>
<tr><td>Cluster 2</td><td colspan="2">6 months CONTROL</td><td colspan="2">6 months INTERVENTION</td></tr>
<tr><td>Cluster 3</td><td colspan="3">9 months CONTROL</td><td>3 months INTERVENTION</td></tr>
</table>

During the control phase, nursing home residents and their families or substitute decision makers will be recruited to complete a survey to explore patient attitudes about deprescribing in this NB study population. The revised Patient Attitude Towards Deprescribing (rPATD) questionnaire “older adult” version will be provided to nursing home residents and the “caregiver” version will be provided to family members and substitute decision makers. Survey questions will be entered in Lime survey to promote electronic completion whenever possible. A recruitment poster will be displayed at study sites and a recruitment communication will be sent to family members and substitute decision makers identified in the resident’s admission package. Paper copies will also be available from the study site contact. Residents and family members who ask nursing home staff about the survey will have their contact information forwarded to the CIRA research manager to answer questions and obtain verbal consent to have a survey e-mailed to them. If e-mail is not available, a research assistant will contact the participant to administer the survey over the phone. Study site contacts may also distribute paper copies of the surveys if requested and permitted by the study site. Stamped envelopes addressed to the CIRA research manager will be provided with each paper survey distributed.

During the control phase, health care providers and study site staff will be recruited to register for MedReviewRx. A recruitment communication will be sent by e-mail and placed in study site

mailboxes. Study recruitment will also be encouraged at interdisciplinary nursing home meetings such as Pharmacy and Therapeutics and Medical Advisory Committee meetings. User training will be provided by the study site contact or designate(s) as determined by the site-specific implementation plan. Nursing home site contacts will participate in an interview to evaluate MedReviewRx implementation. Implementation interviews will be conducted within the first 3 months of the site intervention phase videoconference by the CIRA research manager or designate via telephone or Zoom.

During the intervention phase, MedReviewRx will be made available to nursing homes with the understanding that it will be used to facilitated medication reviews and prescription check-ups. MedReviewRx provides clinicians with access to individualized and prioritized deprescribing information from MedSafer which: a) identifies PIMs, b) explains why the medication is potentially inappropriate and c) provides instructions on how to safely stop/taper the medication. Nursing homes will also have the option to print MedSafer deprescribing reports from MedReviewRx for clinicians who do not have computer access or who choose not to register to use MedReviewRx. If reports are printed, they will be placed in a binder for the prescriber to sign and date indicating that they have read the report. Prescribers and nursing home staff will be encouraged to write feedback directly on the report if they wish too. To assess the proportion of reports that were read, and feedback provided on the reports, signed reports will be kept in a locked cabinet for research assistants to collect. If visitor restrictions are in place, the study site contact will gather signed reports, place them in a sealed envelope(s) and send them to the CIRA research manager using a courier method that can be tracked and requires a sending signature and signature of receipt. Courier costs will be paid for by CIRA.

Clinicians will review deprescribing opportunities (electronically or via a paper report) and determine if medications can be tapered or stopped. Medication changes are discussed with the resident or

substitute decision maker as part of usual care. This process will not change with the implementation of MedReviewRx. If the prescriber decides to alter a medication based on the deprescribing opportunities provided by MedSafer and their expert knowledge, the prescriber will do so in the same manner as they did prior to implementation of MedReviewRx, and discussion with the resident and/or substitute decision maker will occur according to the process in place at each nursing home.
During the intervention phase, an analysis of deprescribing opportunities will be conducted by MedSafer once every 3 months for each resident in the nursing home. Results will be stored in the MedReviewRx system to be accessed at any time.

MedReviewRx has been designed to have Administrator accounts and Clinician accounts which restrict access according to user function. Study site contacts (or site designate) will load data into MedReviewRx using the Administrator function. Prescribers (physicians and nurse practitioners) and pharmacists will be provided with Clinician accounts. Clinicians will only access MedReviewRx records for patients under their care. MedReviewRx registration has been designed to restrict nursing home staff access to residents at their site only. Prescriber and pharmacist access will be restricted to study sites where they practice. MedReviewRx access will be audited by the CIRA research manager or designate.

User feedback on MedReviewRx and MedSafer deprescribing information will be solicited from prescribers, pharmacists and nursing home staff throughout the study using surveys. Surveys may be completed via Lime Survey, on paper or by telephone (depending on the preference of the person). Three surveys will be distributed by e-mail to registered MedReviewRx users as well as other study site prescribers, pharmacists and nurses who may have access to printed reports from MedReviewRx. Paper copies of the surveys will also be available at the study site nursing units. A MedReviewRx user

feedback survey will be distributed at the end of the first and third quarter of the intervention and an acceptability and feasibility survey will be distributed at the end of the study. Paper copies of completed surveys will be collected by the study site contact, placed in a sealed envelope(s) and sent to the CIRA research manager using a courier method that can be tracked and requires a sending signature and signature of receipt. Courier costs will be paid for by CIRA. MedReviewRx users will be provided with a study e-mail which they may use to submit feedback and questions at any time throughout the study. Informal feedback received by e-mail from users will be reviewed and actioned by the CIRA research manager or designate. E-mail feedback will be documented anonymously in an excel spreadsheet and reported to the study principle investigators within 1 week of receipt to determine if tasks need to be submitted to the technical team for software updates. Rapid cycle improvements in usability of the system and MedSafer output will be made based on survey results and informal feedback.
Informal feedback written on printed MedSafer reports will be documented in an excel spreadsheet and included in the analysis of qualitative feedback provided by survey and interview.

Frequency of MedReviewRx use will be measured electronically by counting the number of times a user accesses their account.

Overall experience with the research project will be evaluated using semi-structured interview conducted at the end of the intervention period.

Sampling procedures

Convenience sampling was used to select nursing homes for the study. New Brunswick nursing home administrators and directors of nursing were contacted by e-mail to determine interest in study participation.

Convenience sampling will also be used to recruit health care providers, older adults and family members of older adults from study nursing homes. A recruitment poster for resident and family member surveys will posted in study nursing homes and a communication will be sent to families and substitute decision makers by regular mail or e-mail using the study site contact list. Health care providers and study site staff will be recruited to register for MedReviewRx by placing communication in their nursing home site mailbox and/or by e-mail. All pharmacists, nurses, nurse practitioners and physicians providing care at the participating nursing home will receive recruitment e-mails for MedReviewRx feedback surveys. Members of healthcare teams at the study sites will be recruited to participate in interviews to explore overall experience with the research project. Recruitment communication for the End of Study Feedback Interview will be sent by e-mail and placed in study mailboxes.

Variables under investigation include:

a) Patient demographics (date of admission to LTC, age, sex, nursing home identifier, medical and psychiatric conditions documented in the interRAI LTCF assessment, and date of discharge from LTC if applicable,

b) Medication use data: absolute number of medications, number of PIMs, proportion of all medications that are PIMs, and type of deprescribing opportunities identified.

c) Safety data: death, fracture, fall, use of restraints, transfer to hospital, pressure ulcers and stage, in-dwelling catheter use, change in functional status based on interRAI activities of daily living (ADL) scores, and transfer out of the institution (return to independent living). Episodes of delirium based on

responses to questions in Section C of the interRAI LTCF assessment form (periodic disordered thinking or awareness and acute change in mental status from person's usual functioning).

d) Health care provider experience with MedReviewRx and MedSafer deprescribing information as described in the user feedback surveys and the acceptability and feasibility survey.

e) Nursing Home evaluation of the implementation toolkit

f) Patient and caregiver attitudes about deprescribing

Data collection

Patient demographics and safety data is entered in the interRAI LTCF assessment software by nursing home staff as part of usual care. Medication information will be obtained from nursing home medication administration records (MARs) and entered in section N1 (list of all medications) of the interRAI LTCF assessment software by nursing home staff or a trained research assistant. Research assistants who enter medication data will receive training on how to enter medication information into Momentum LTCF assessment software. This training will occur at the CIRA research office in Fredericton, or virtually by Zoom. If onsite visits are permitted, the research assistant will also receive orientation to the nursing home sites. If onsite data entry is not permitted due to COVID precautions, medication data will be entered remotely at the CIRA research office in Fredericton via a secure remote access portal into the Momentum LTCF assessment software for the study site. Remote portal access will be set up using VPN access in collaboration with the study site. Capability for remote access to specific nursing home sites currently exists for the NB Momentum administrator and will be requested for research assistants if required. All research assistants will undergo training on privacy and confidentiality in accordance with CIRA policies and procedures.

Patient demographics, medication use, and safety data will be extracted from the LTCF interRAI assessment software and loaded into MedReviewRx by the study site contact (or designate) at each nursing home. The nursing home contact (or designate) will be provided with written instructions and receive a demonstration on how to perform this function. They will also receive a telephone number for a research team member who can assist them with questions or concerns for the duration of the study.

MedReviewRx will deidentify the data and transmit data sets to MedSafer. Multiple safeguards have been put in place to protect this data. Date sets do not contain patient specific information. Age is transmitted rather than date of birth, and a unique identifier is assigned to each patient data set. MedSafer is unable to identify patients however the unique identifier allows MedReviewRx and MedSafer applications to anonymously communicate about specific patients through an API. Data sets will be analyzed by the MedSafer software program. Files containing one or more targeted PIM and associated triggering condition(s) will have deprescribing opportunities flagged and returned to MedReviewRx. MedReviewRx will link the unique identifier to the correct nursing home patient and translate the MedSafer analysis into an output for review by their health care team. A message will also be displayed by MedReviewRx if there are no deprescribing opportunities identified for a data set. MedReviewRx transmits and stores data using a secure web-based system which was designed by Missing Links Technology under the supervision of Dr William McIver Jr, NSERC Industrial Research Chair, Mobile First Technology Initiative at the New Brunswick Community College. The MedReviewRx system safeguards personal health information (PHI) that it collects, processes and disseminates within trusted and authorized circles of care and ensures the confidentiality and privacy of the PHI. Each study site using MedReviewRx has site specific policies and procedures with respect to privacy and confidentiality of PHI.

Data from User Feedback Surveys and Acceptability and Feasibility Surveys and survey responses collected from the revised Patient Attitude Towards Deprescribing (rPATD) questionnaires completed via Lime Survey will be extracted into excel sheet format (an excel sheet for each type of survey). Survey data collected from paper surveys or telephone surveys will be entered into the excel database by a research assistant. Research assistants will be provided with templates for excel data entry and trained on how to complete the template. A minimum of 5% of the research assistant data entry will be audited for accuracy and completeness by the CIRA research manager or designate. Interview data will be recorded and transcribed verbatim by research assistants. Interview recordings will be deleted after transcripts have been validated. Study data (PIMs, survey responses, interview transcripts) will be stored in password protected, members only secure cloud-based study site hosted within Canada. Study data will only be accessible to the study research team and the CIRA research manager. On study closure, after final data analysis is completed, study data will be downloaded to a password protected study folder on the YCC secure network and the cloud-based study site will be permanently deleted. Study data will be stored for 7 years and then destroyed in accordance with YCC/CIRA policies and procedures.

Data Analysis Plan

This mixed methods study will employ both qualitative and quantitative data analysis. The primary outcome of interest is: the impact of MedReviewRx on the prevalence of PIMs in NBNHs

Study patient demographics (age, sex, gender, geographic location, common medical conditions, common PIMs) will be described descriptively.

<u>Impact on PIMs</u>

The impact on PIMs will be determined by the proportion of nursing home residents who have one or more PIMs reduced or stopped during the intervention as compared to the control. This outcome will be measured by using unique identifiers to compare medication data from sequential interRAI data sets. PIMs identified in the baseline data set, will be compared with the next data set (3 months later) transmitted with that unique identifier to determine if any of the PIMs has been stopped (no longer listed) or if a PIM dose has been reduced. This process will continue for the study duration. A marginal generalized estimating equation (GEE) model will be used controlling for site clustering and repeated measurements as random effects, and age, sex, and language as fixed effects. All analyses will be conducted using SAS 9.4 (SAS Institute, Inc., Cary, North Carolina, USA).

Power calculation

At baseline, a reasonable estimate of deprescribing based on usual care is that about 20% of residents have one or more PIMs deprescribed following a medication review. Based on prior studies, conservatively we estimate that we could increase this by 20% to 40% by providing a MedSafer report via the MedReviewRx. With approximately 250 nursing home residents per cluster (min of 750 residents) this population gives us at least 80% power with 5% alpha to detect a 20% absolute increase in deprescribing. For the primary outcome, deprescribing 1 or more PIM, a marginal generalized estimating equation (GEE) model will be used controlling for site clustering and repeated measurements as random effects, and age, sex, and language as fixed effects. Odds ratios (OR) and 95% confidence intervals (CIs) will be estimated from the model parameters. This analysis will be restricted to data from residents who were taking at least 1 PIM. For secondary outcomes, an identical analysis will be conducted. All analyses will be conducted using SAS 9.4 (SAS Institute, Inc., Cary, North Carolina, USA) or similar.

Secondary outcomes

Secondary outcomes will include falls, use of restraints, episodes of delirium based on responses to questions in Section C of the interRAI LTCF assessment form (periodic disordered thinking or awareness and acute change in mental status from person's usual functioning). We will not explore death as the cohort needs to survive 3 months to receive the intervention leading to an imbalance in deaths between control and intervention.

Tertiary outcomes

User experience

User experience with MedSafer reports and MedReviewRx will be measured using survey responses as well as described through interview data and informal feedback received by the study team. Several outcomes will be studied: how satisfied the user is with the App, including how helpful they find it, the added knowledge from a MedSafer report for the prescriber, how likely the prescriber is to continue using the App once the study is over. Survey responses will be analyzed based on the following themes: Value & benefits, scientific content, and user satisfaction. Interview data will be thematically analysed (Braun & Clarke, 2006).

A cost savings analysis

Related to cost saved from medications (actual price of the medication as well as dispensing fees) balanced with the cost of deployment of MedSafer including maintaining the program with updates and user support.

Patient and family attitudes about deprescribing

Reported using survey responses collected from the revised Patient Attitude Towards Deprescribing (rPATD) questionnaires.

Project evaluation

This project will be evaluated locally at each participating nursing home site to determine usability, impact on workflow, time commitment, and value added. Qualitative and quantitative user feedback as described above will be used to explain study findings.

Patient and family attitudes about deprescribing will be described descriptively using survey responses collected from the revised Patient Attitude Towards Deprescribing (rPATD) questionnaires.

An analysis will be conducted to determine strategies to inform widespread deployment, nursing home evaluation of the implementation toolkit, prescribing patterns based on geographic area and overall change in PIM use. System level impact on cost-savings and cost avoidance of adverse effects will be projected.

Conclusion

The goal of this project is to study the impact of MedReviewRx, an intervention designed to help address the issue of medication overload among older adults living in nursing homes in New

Brunswick. MedReviewRx allows health care providers (prescribers, pharmacists and nurses) in nursing homes to access MedSafer analysis on a tablet or laptop. This mixed-methods study has been designed to measure the baseline prevalence of PIMs in study nursing homes and to calculate the change in PIM use after MedReviewRx is implemented. User experience with MedSafer reports and MedReviewRx will also be explored as well as nursing home evaluation of the implementation process and patient and family attitudes about deprescribing. This study will provide valuable information on PIM use as well as facilitators and challenges associated with medication reviews and deprescribing. We hope that this study will demonstrate that implementing MedReviewRx is feasible and acceptable to health care providers in NBNHs and that it results in a decreased PIMs and cost savings. This study is an important step towards understanding and promoting tools to guide safe and rational reduction in PIM use among older adults.

References

AlRasheed MM, Alhawassi TM, Alanazi A et al. Knowledge and willingness of physicians about deprescribing among older patients: a qualitative study. Clin Interv Aging. 2018 Aug 6;13:1401-1408.

Braun, V., & Clarke, V. (2006). Using thematic analysis in psychology. Qualitative research in psychology, 3(2), 77-101.

Brath et al. What is known about Preventing, Detecting and Reversing Prescribing Cascades: A Scoping Review. J Am Geriatr Soc. 2018; 66:2079-2085.

Canadian Institute for Health Information. Drug Use Among Seniors in Canada, 2016. Ottawa, ON: CIHI; 2018.

Conklin J, Farrell B, Suleman S.Implementing deprescribing guidelines into frontline practice: Barriers and facilitators. Res Social Adm Pharm. 2019 Jun;15(6):796-800. doi: 10.1016/j.sapharm.2018.08.012. Epub 2018 Sep 18.

Dills H, Shah K, Messinger-Rapport B, Bradford K, Syed Q. Deprescribing Medications for Chronic Diseases Management in Primary Care Settings: A Systematic Review of Randomized Controlled Trials. *JAMDA.* 2018;19: 923e935.

Djatche L, Lee S, Singer D et al. How confident are physicians in deprescribing for the elderly and what barriers prevent deprescribing? J Clin Pharm Ther. 2018 Aug;43(4):550-555.

McDonald EG, Wu PE, Rashidi B et al, The MedSafer Study: A Controlled Trial of an Electronic Decision Support Tool for Deprescribing in Acute Care. J Am Geriatr Soc. 2019 Sep;67(9):1843-1850.

Sirois C, Ouellet N, Reeve E. Community-dwelling older people's attitudes towards deprescribing in Canada. Res Social Adm Pharm. 2017;13(4):864-870.

Thillainadesan J, Gnjidic D, Green S, Hilmer S. Impact of Deprescribing Interventions in Older Hospitalised Patients on Prescribing and Clinical Outcomes: A Systematic Review of Randomised Trials. *Drugs Aging.* 2018; 35:303–319.

Appendix A -MedSafer Report Output

Page 1

Deprescribing Opportunities as of 2018-02-06

This document contains prioritized opportunities for a reassessment of the listed medications. Any decisions should take into context what you know about your patient and your clinical assessment of the risks and benefits of what has been presented.

Duck Jr., Donald M (Mr.)
York Care Centre
Medicare No.:
Born: 1930-03-15 (Age: 87)
Admission: 2016-02-14

TI - Tapering instructions or withdrawal concerns? / Please refer to to tapering instructions on last page.

Condition/Drug	Cause of Alert	Why might this be inappropriate?	TI
**Drugs Considered Intermediate Risk for Adverse Drug Event**			
gabapentin (Mylan-gabapentin)	Any	Pregabalin and gabapentin may lead to peripheral edema, impaired cognition, confusion and falls. The evidence for use outside of diabetic neuropathy and zoster is limited.	Yes
pantoprazole (Apo-pantoprazole)	Any	Chronic PPI therapy should be re-evaluated regularly. For patients aged 60 years and older along with two or more of the following, ongoing therapy may be beneficial: antiplatelet, NSAID, systemic steroids, anticoagulation, prior upper gastrointestinal bleed. Other scenarios requiring ongoing therapy include: hypersecretory conditions, dual antiplatelet therapy, variceal banding within 14 days, and H. Pylori treatment.	Yes

Appendix A -MedSafer Report Output

Page 2

Deprescribing Opportunities as of 2018-02-06

Tapering Instructions

gabapentin (Mylan-gabapentin) Cause of Alert: Any

Tapering required. Reduce dose by 25 to 50% every 1 to 2 weeks. Dose reductions may need to slow down at smaller doses (ex. 25% of original dose).

pantoprazole (Apo-pantoprazole) Cause of Alert: Any

Stopping a PPI abruptly may lead to rebound hyperacidity. Patients on long term therapy (>3 months) or high doses may benefit from tapering over 2 to 4 weeks. Lowering the PPI dose (for example: from twice daily to once daily, halving the dose when possible, or taking every second day) OR changing from regular use to as needed are equally acceptable approaches.

Appendix B: Polypharmacy App Screenshots

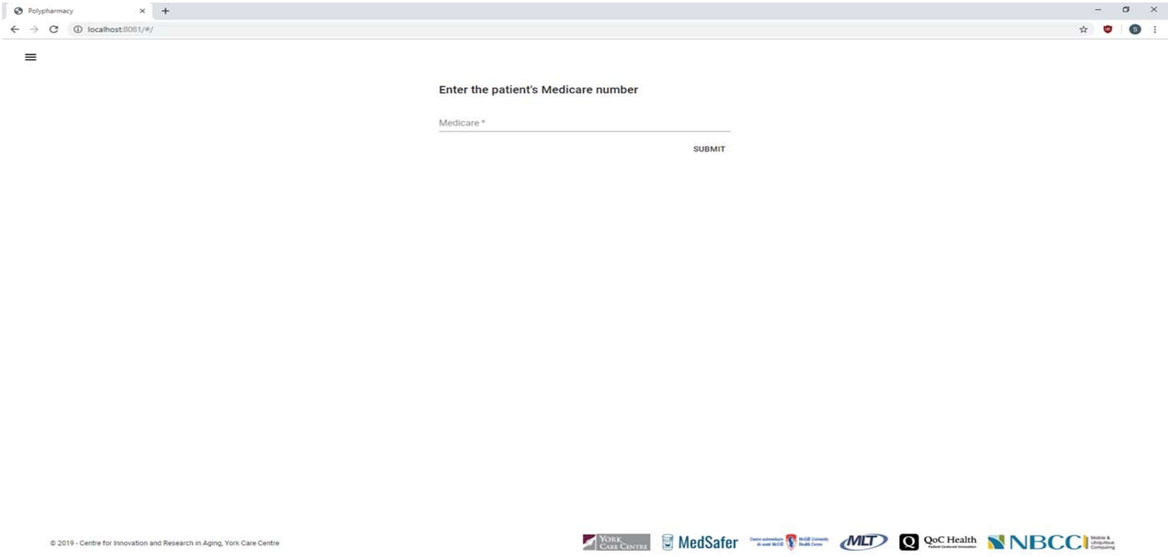

Appendix B: Polypharmacy App Screenshots

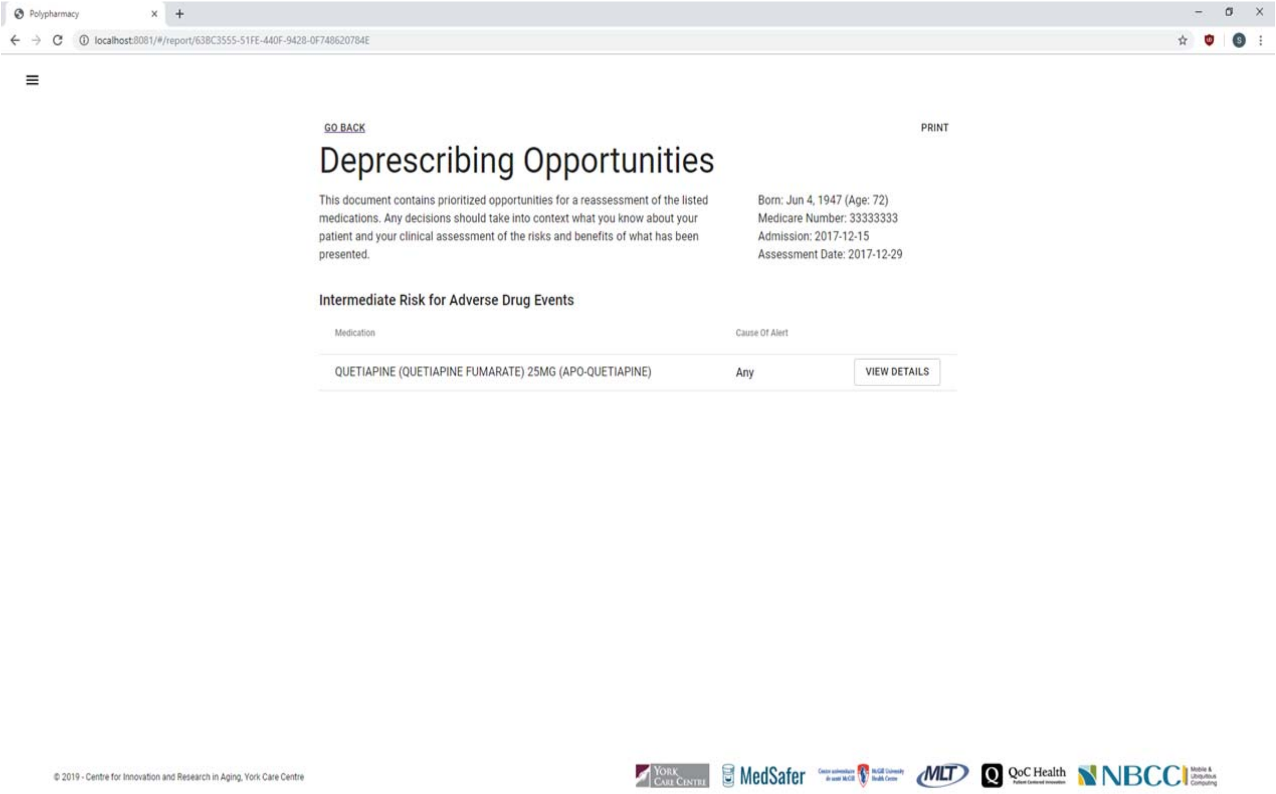

Appendix B: Polypharmacy App Screenshots

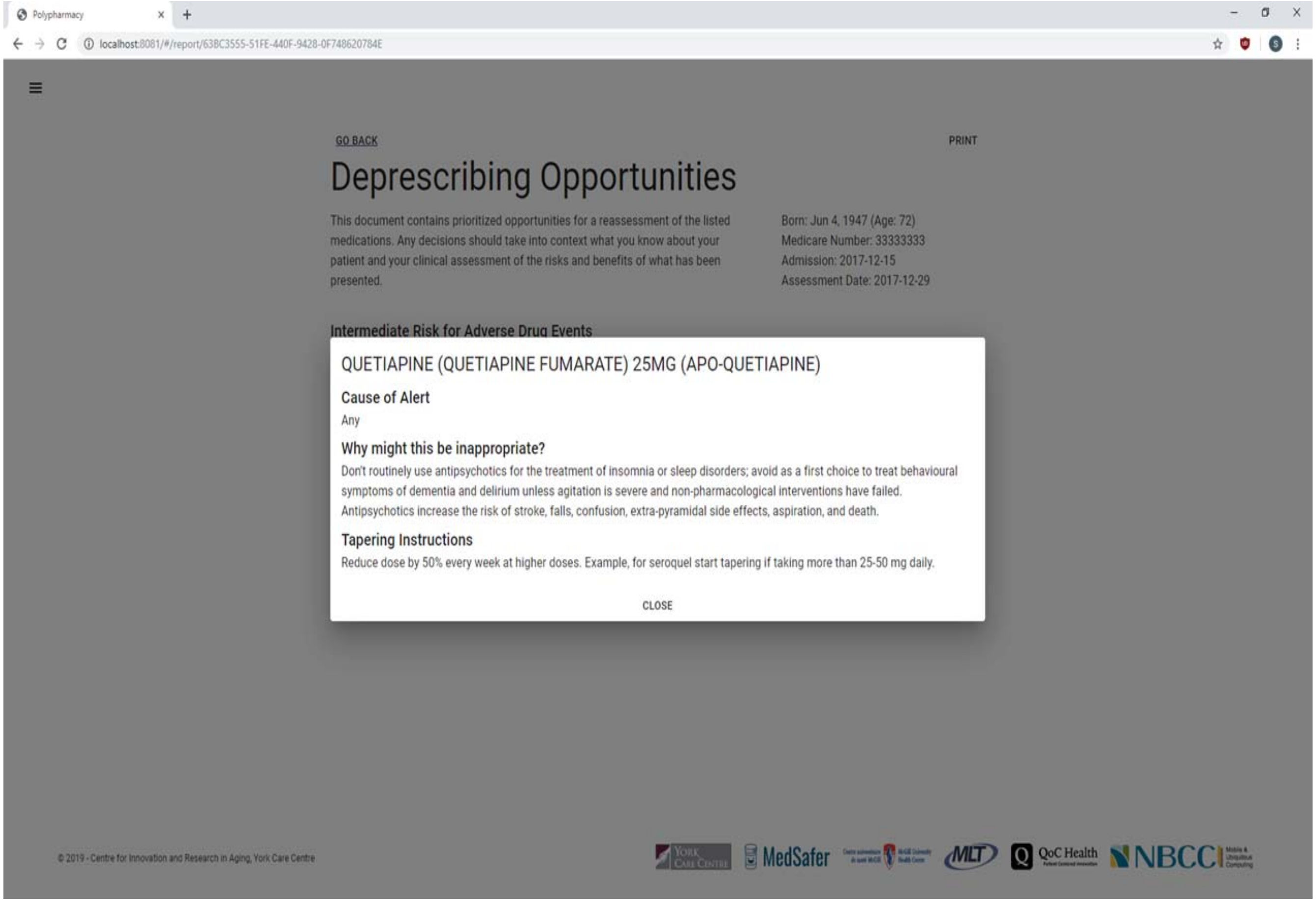

Use the following steps to request *MedSafer Deprescribing Opportunities Reports*:

(1) Choose the `.csv file` containing exported *LTCF records* that you wish to import into the *Polypharmacy System*. This is depicted in the following view.

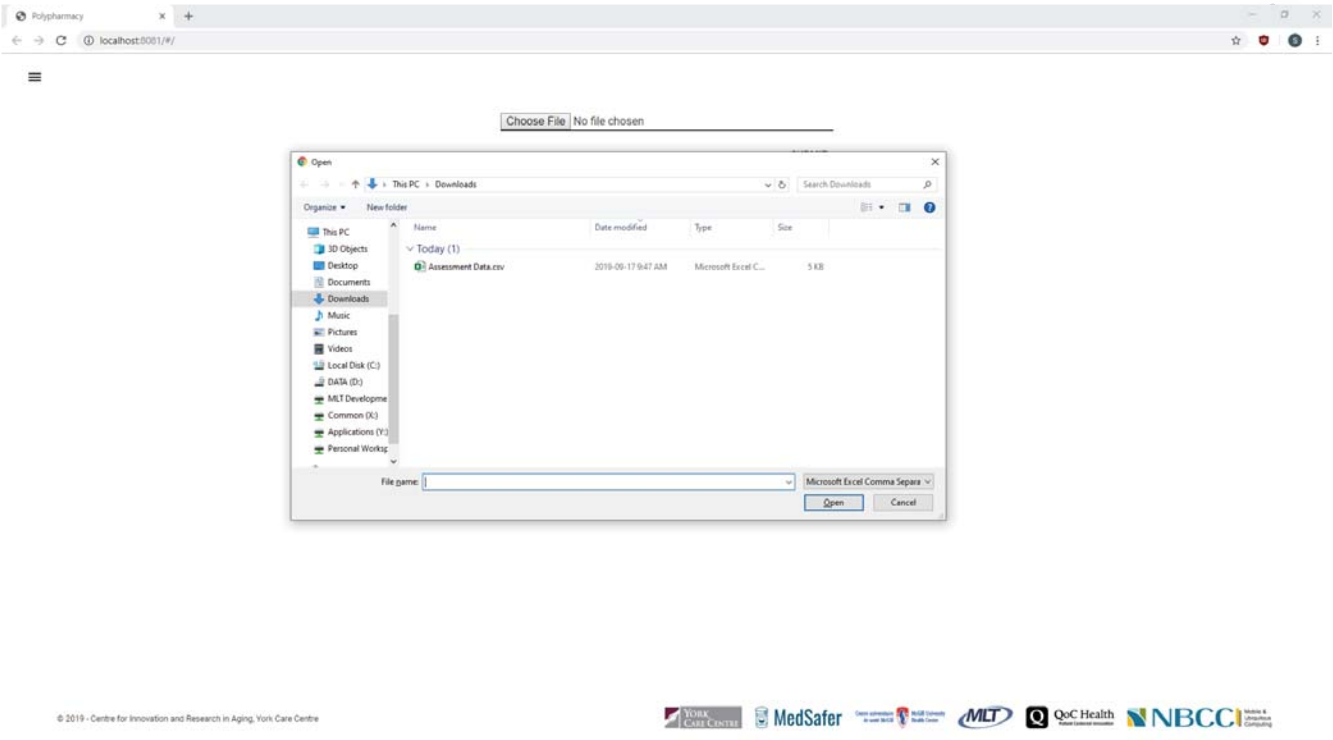

Figure 4. Choosing a `.csv file` containing LTCF records to import in the *Polypharmacy System*.

(2) Confirm your file selection by clicking the OPEN control in the file chooser (or equivalent for your operating system).

DRAFT

(3) After a `.csv file` has been selected, the following view will be seen:

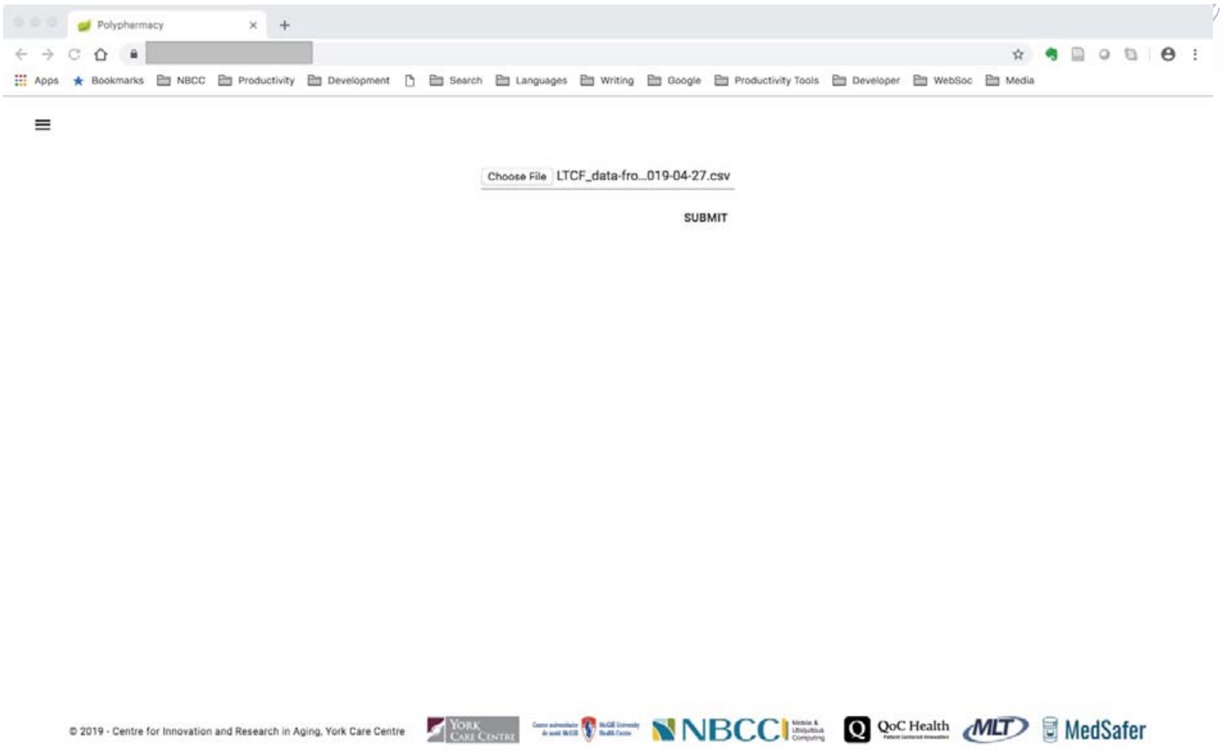

Figure 5. After selecting a `.csv file` containing LTCF records to import.

(4) Click the SUBMIT button to initiate the import process.

DRAFT — COPY

(5) The following view appear after LTCF records have been imported successfully into the *Polypharmacy System,* which results in a *MedSafer Deprescribing Opportunities Reports* being produced for each LTCF record .

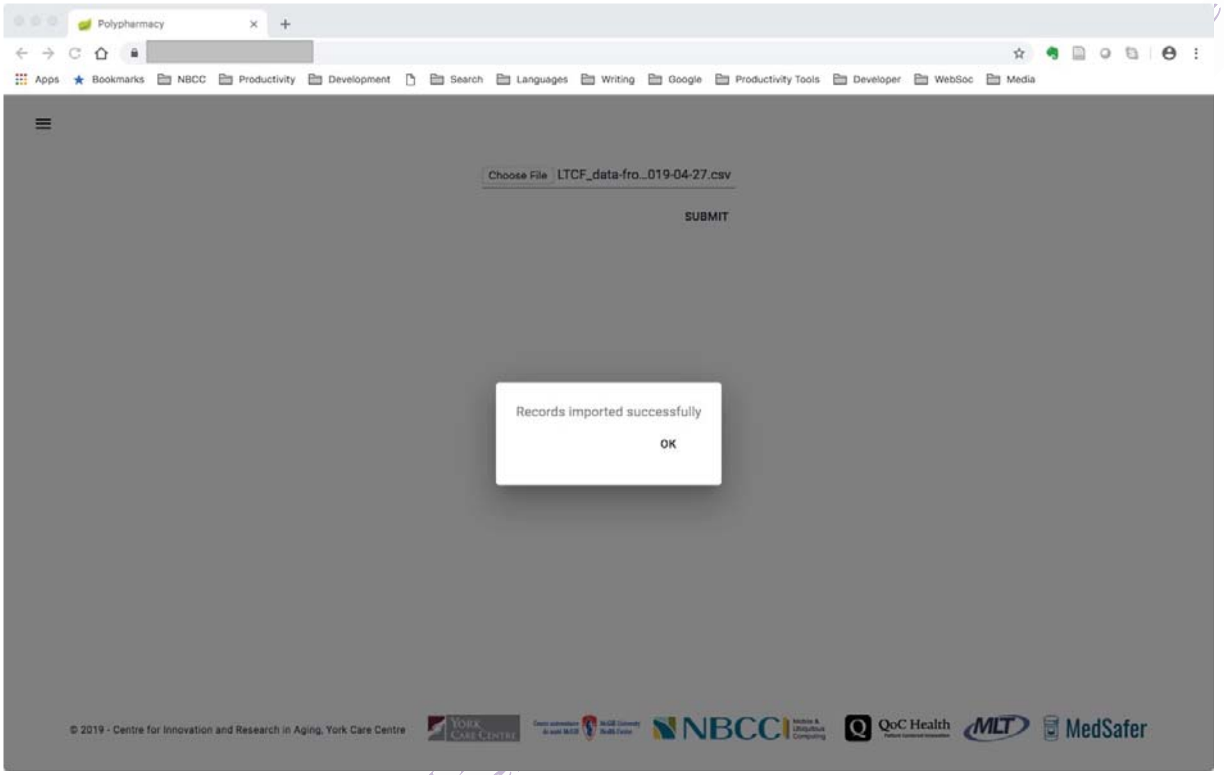

Figure 6. After successful importation of a `.csv file` containing LTCF records.

The importation of a `.csv file` containing LTCF records into the Polypharmacy system causes the following to happen:

a. Each LTCF record in the selected `.csv file` is imported into the *Polypharmacy System*.
b. Each LTCF record is transformed into a special format necessary for requesting a *MedSafer Deprescribing Opportunities Report*, including de-identification of the patient and the use of a persistent, anonymous identifier for the patient to enable *MedSafer's* rules and algorithms to reference longitudinal data from deprescribing reports it has produced in the past about the same patient.
c. A request is sent to *MedSafer* using each newly-transformed LTCF record that has been imported.
d. The *Polypharmacy System* receives and stores each *MedSafer Deprescribing Opportunities Report* in HIPPA and PHIPA-compliant system.
e. The *MedReviewRx App* makes each *MedSafer Deprescribing Opportunities Report* available for review in both the *Admin UI* and *Clinician UI*.

DRAFT

(2) The *Admin UI* can then be used to search for any *MedSafer Deprescribing Opportunities Reports* stored in the *Polypharmacy System* – just now or during past imports. A patient's *Assessment ID* is required to search for reports. This is depicted in the view below.

> Please see section 0 for information about the Assessment ID. Note: The *Clinician UI* allows the user to search for recommendations using a patient's Medicare Number.

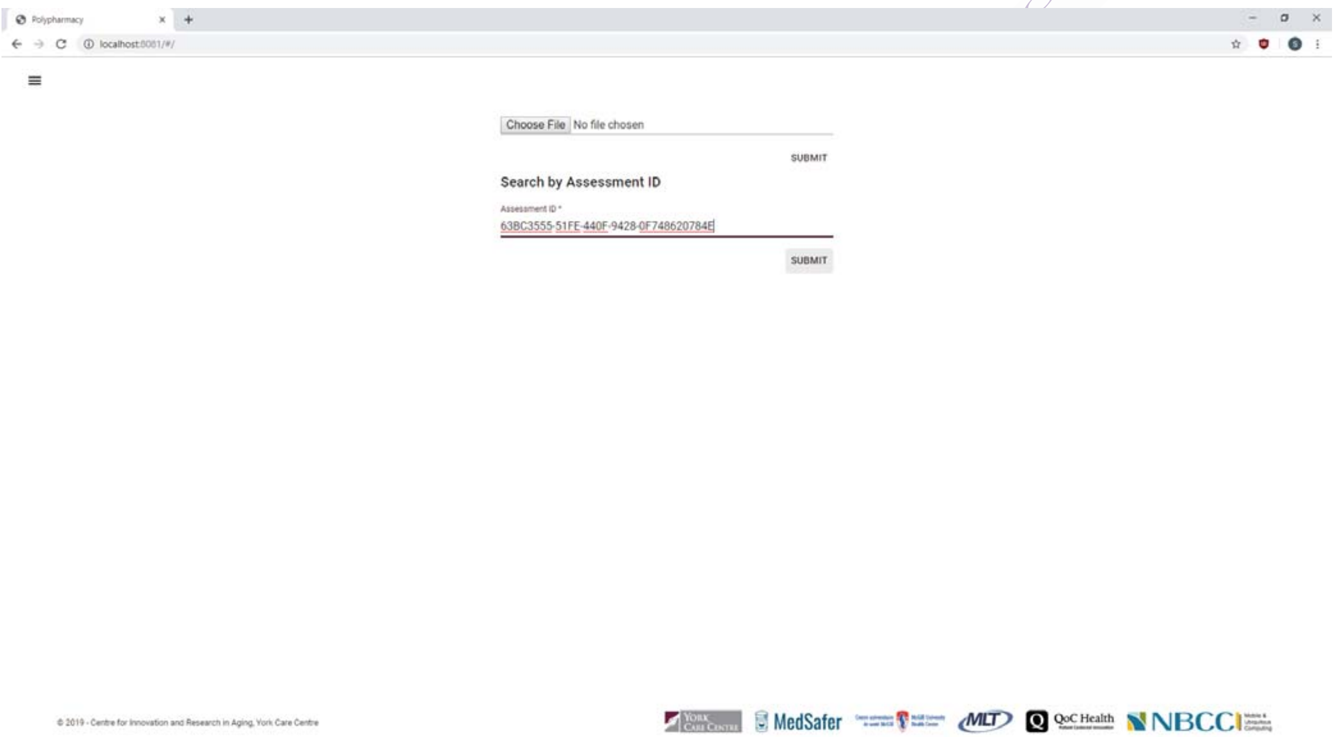

Figure 7. Using *Assessment ID* to search for a *MedSafer Deprescribing Opportunities Report* in the *Admin UI*.

(3) If a *MedSafer Deprescribing Opportunities Report* associated with the *Assessment ID* you have entered is located, it will be made available for review as shown below.

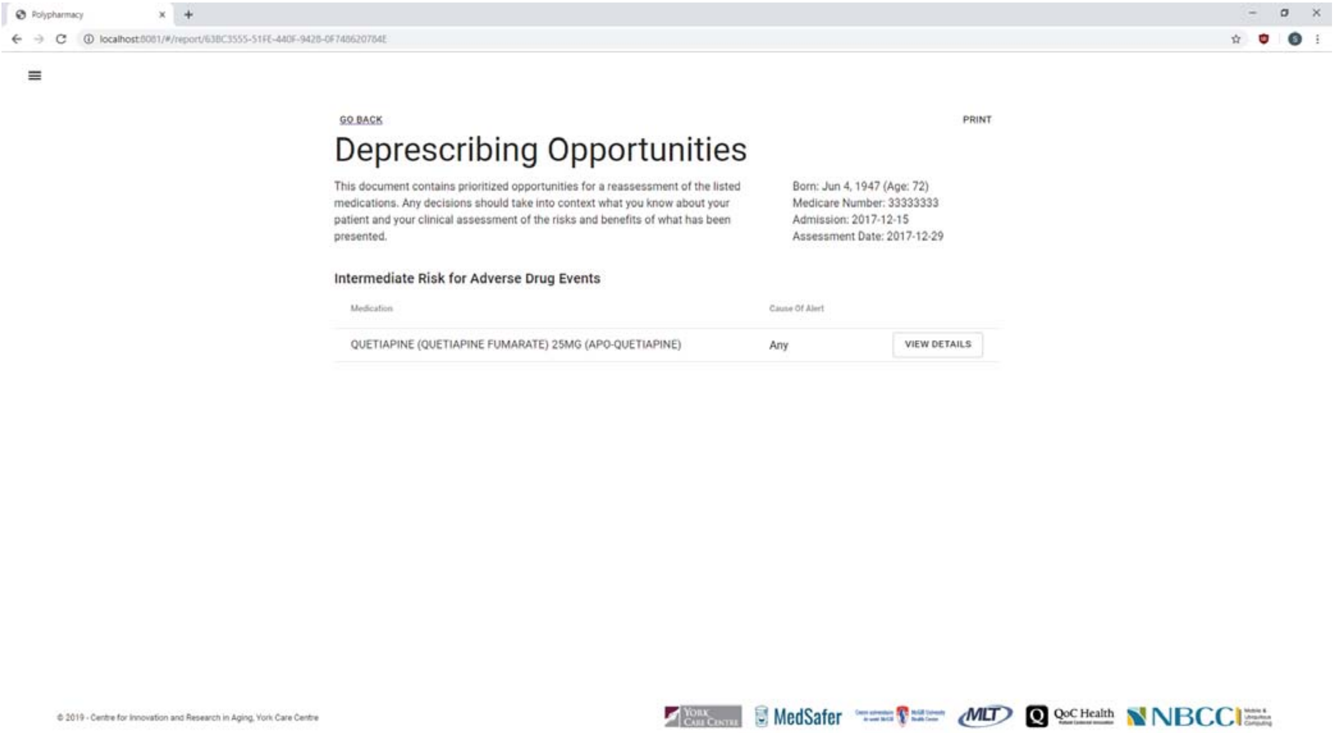

Figure 8. A *MedSafer Deprescribing Opportunities Report*.

(4) Each *MedSafer Deprescribing Opportunities Report* offers additional details. Click the VIEW DETAILS button to view them. This is depicted in the following view.

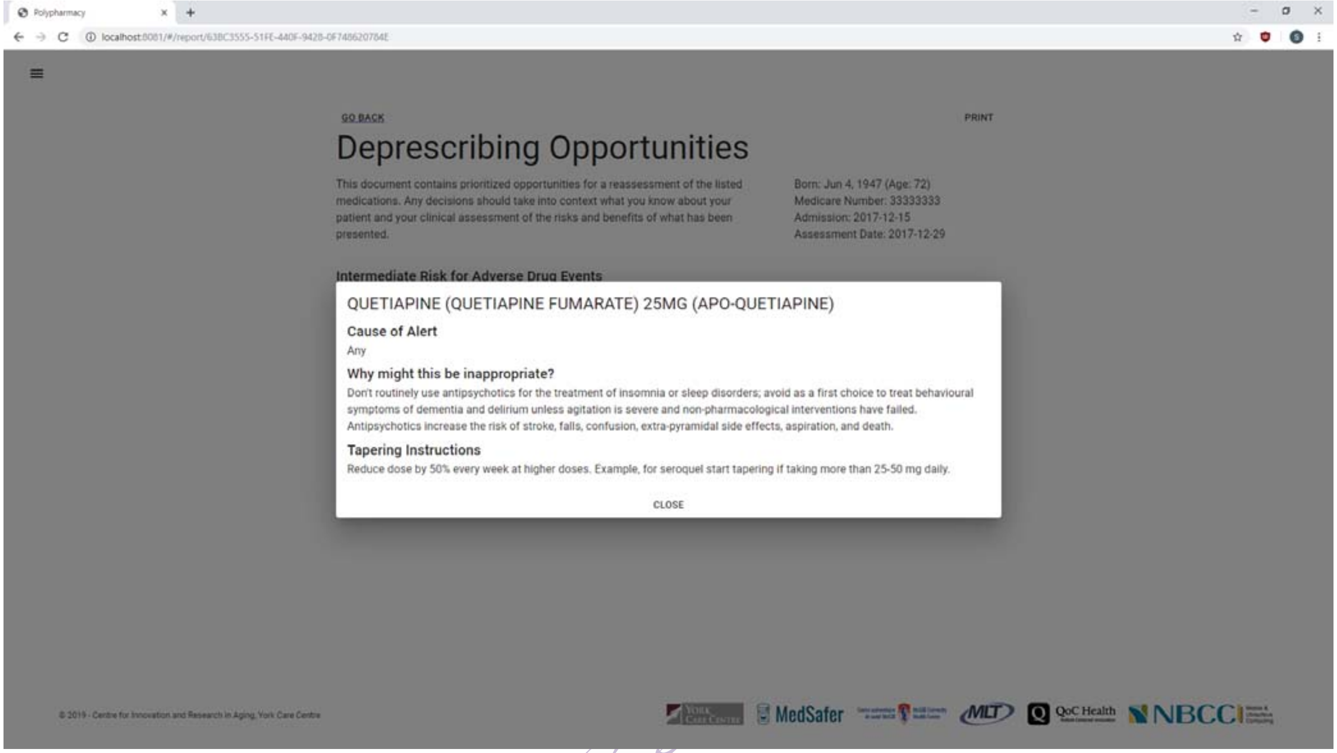

Figure 9. Viewing details within a *MedSafer Deprescribing Opportunities Report*.

(5) CLICK on the PRINT button at the top right of the view to save the *MedSafer Deprescribing Opportunities Report* to a PDF file or to send it to a printer. This is depicted in the following view.

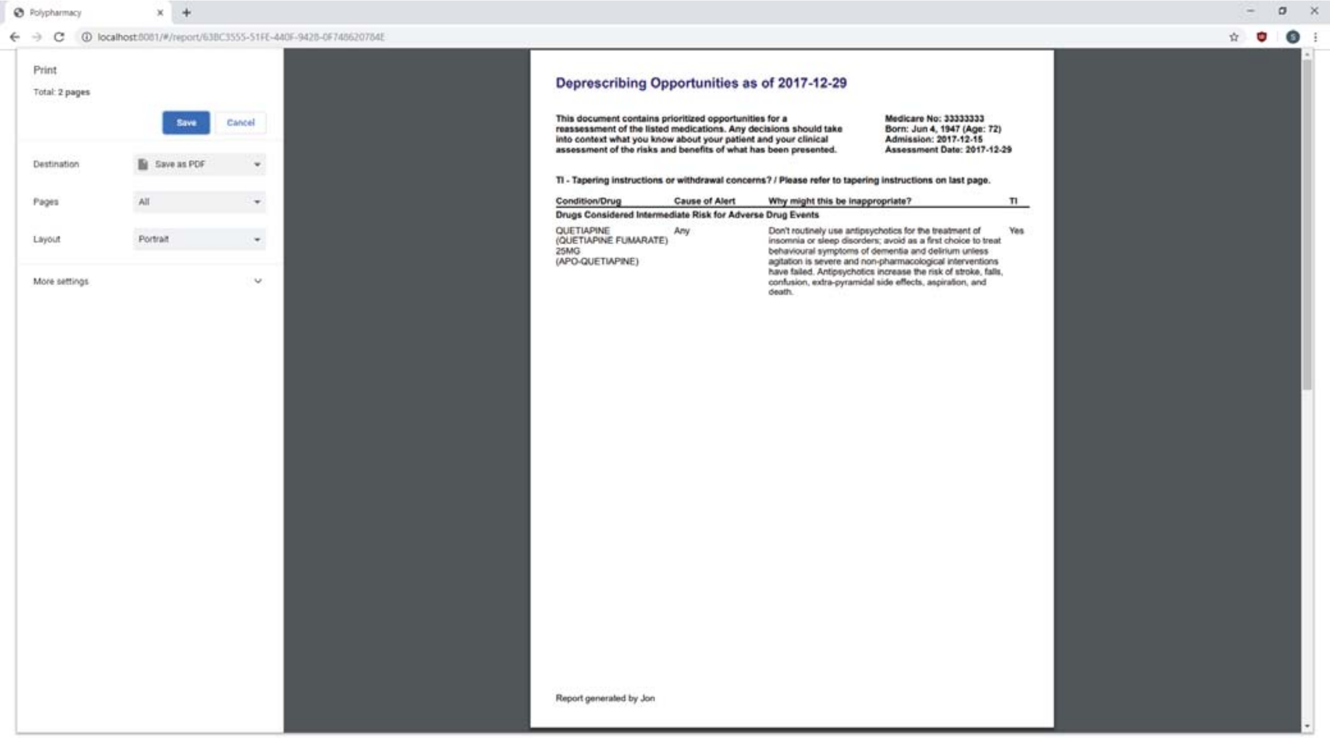

Figure 10. Print a *MedSafer Deprescribing Opportunities Report*.

DRAFT – CONFI
Cent

(6) The tasks explained above may be repeated any number of times for the same or different sets of LTCF reports.
(7) Log out of the *Admin UI* of the *MedReviewRx App* when all tasks have been completed by opening the MENU on the left side of the view and then clicking *Log out.* This is depicted in the following view.

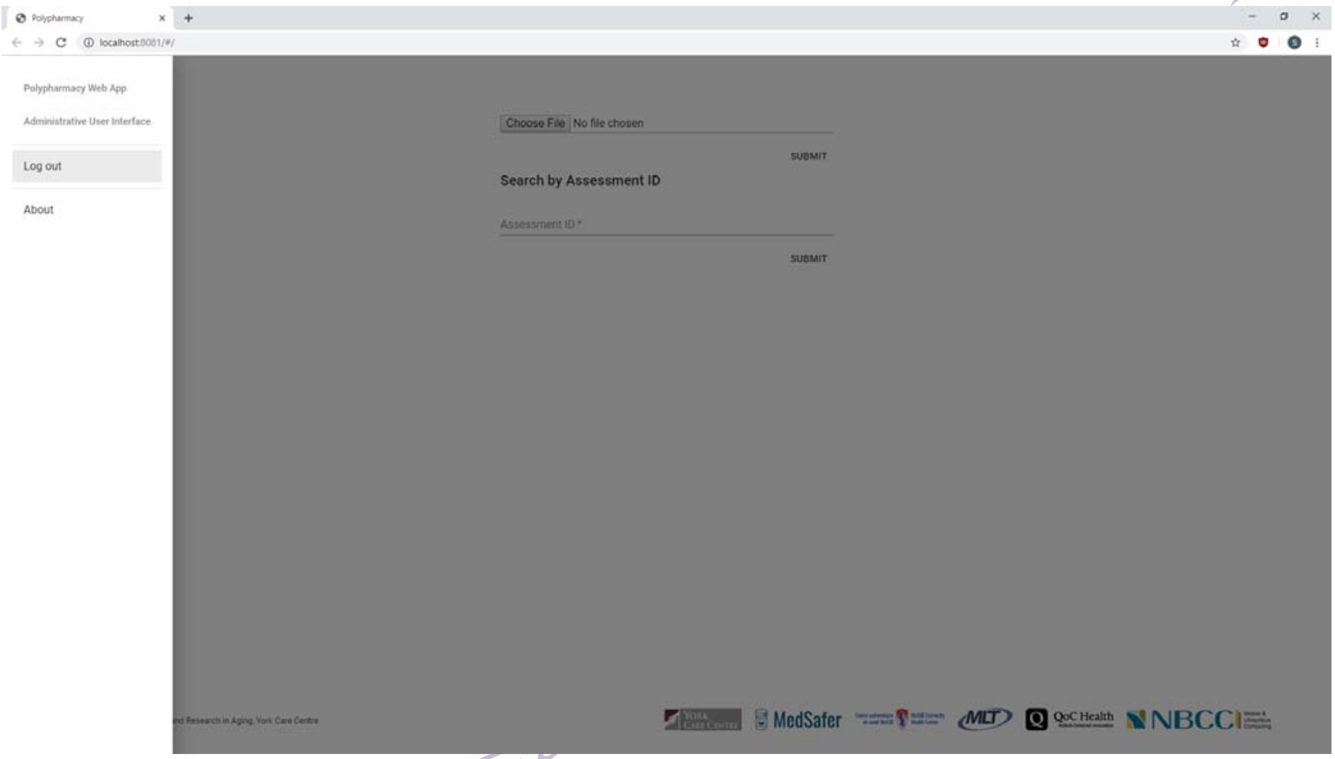

Figure 11. Logging out of the *MedReviewRx App*.

(8) The following view will then be presented.

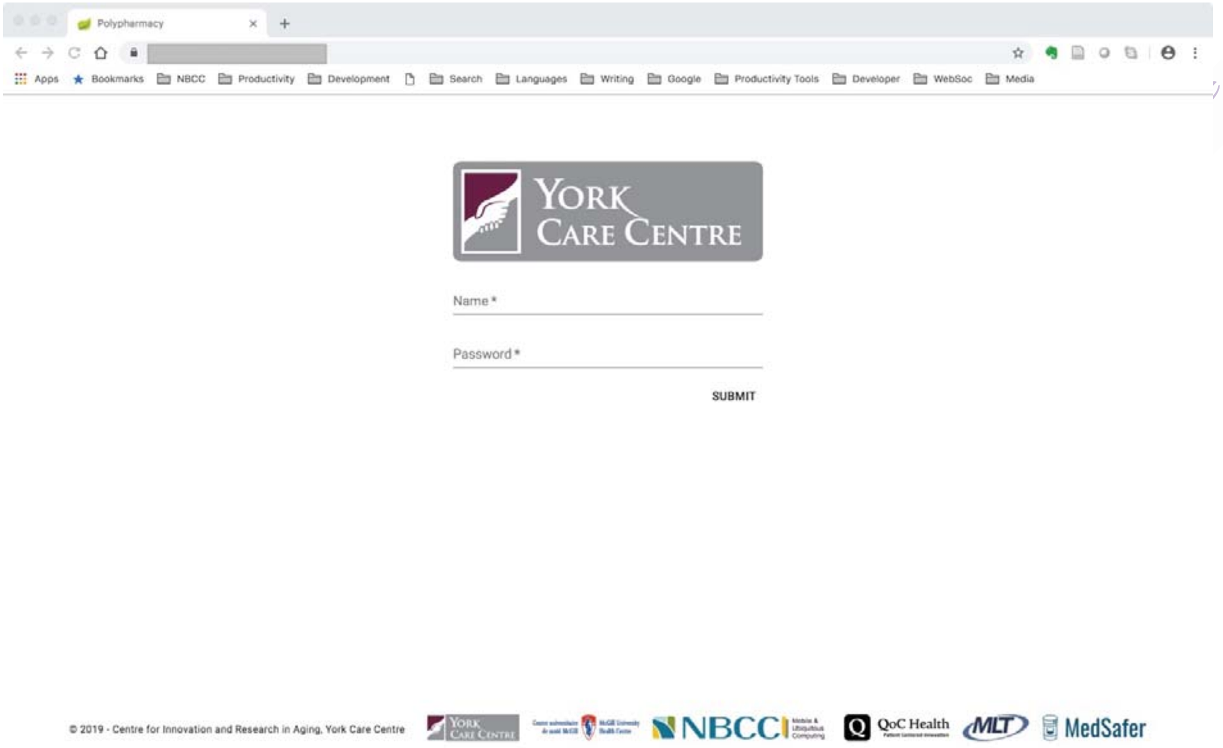

Figure 12. After logging out of the *MedReviewRx App*.

6 USING THE POLYPHARMACY SYSTEM IN THE CLINICIAN ROLE

The Clinician Role is used to search for and view *MedSafer Deprescribing Opportunities Reports* after their associated LTCF records that have been imported in the *Polypharmacy System* by an administrator, as discussed in *section 5*.

(1) Authenticate to the *MedReviewRx App*, as explained in *section 4*.
(2) After authenticating to the *Clinician Role* and entering your name. The Clinical UI then offers the opportunity to locate *MedSafer Deprescribing Opportunities Reports* by entering a patient's Medicare Number. This is depicted below.

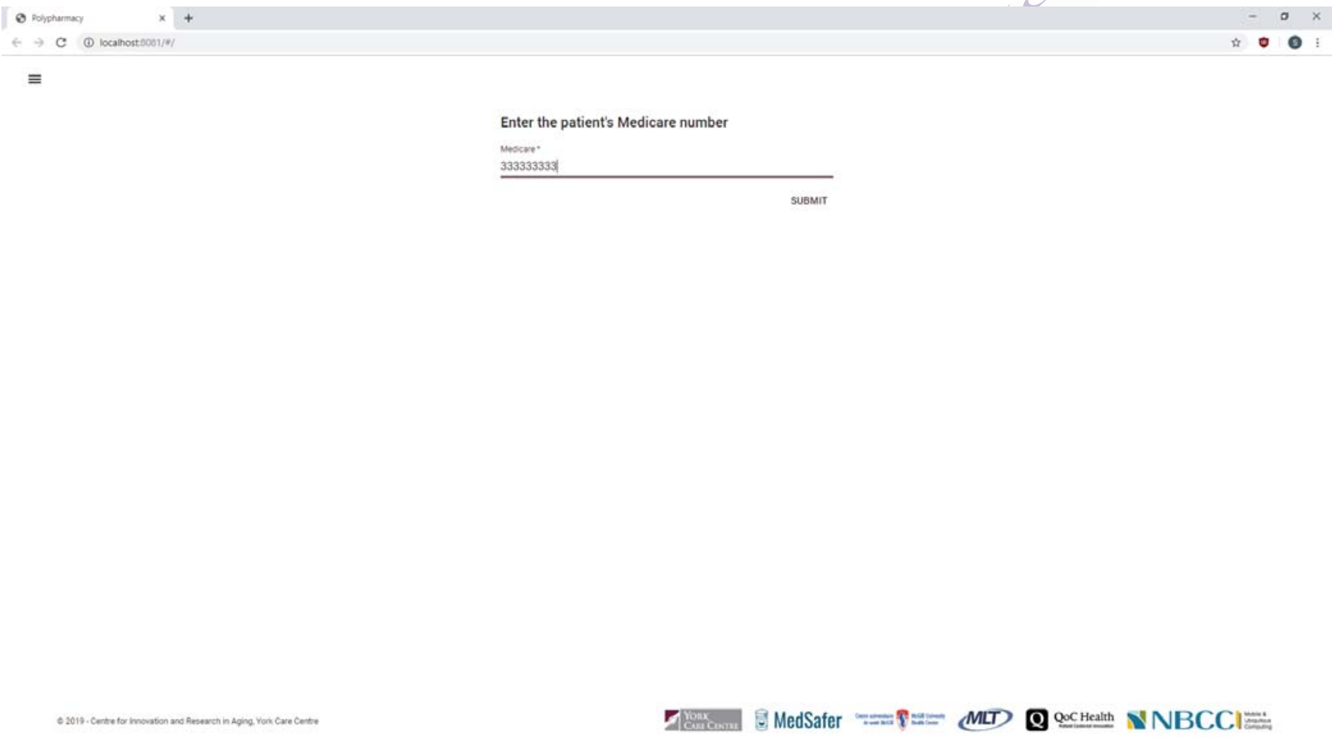

Figure 13. After authenticating to the *Clinician Role*.

DRAFT

(3) If *MedSafer Deprescribing Opportunities Reports* for the patient are located, they will be listed in the following view.

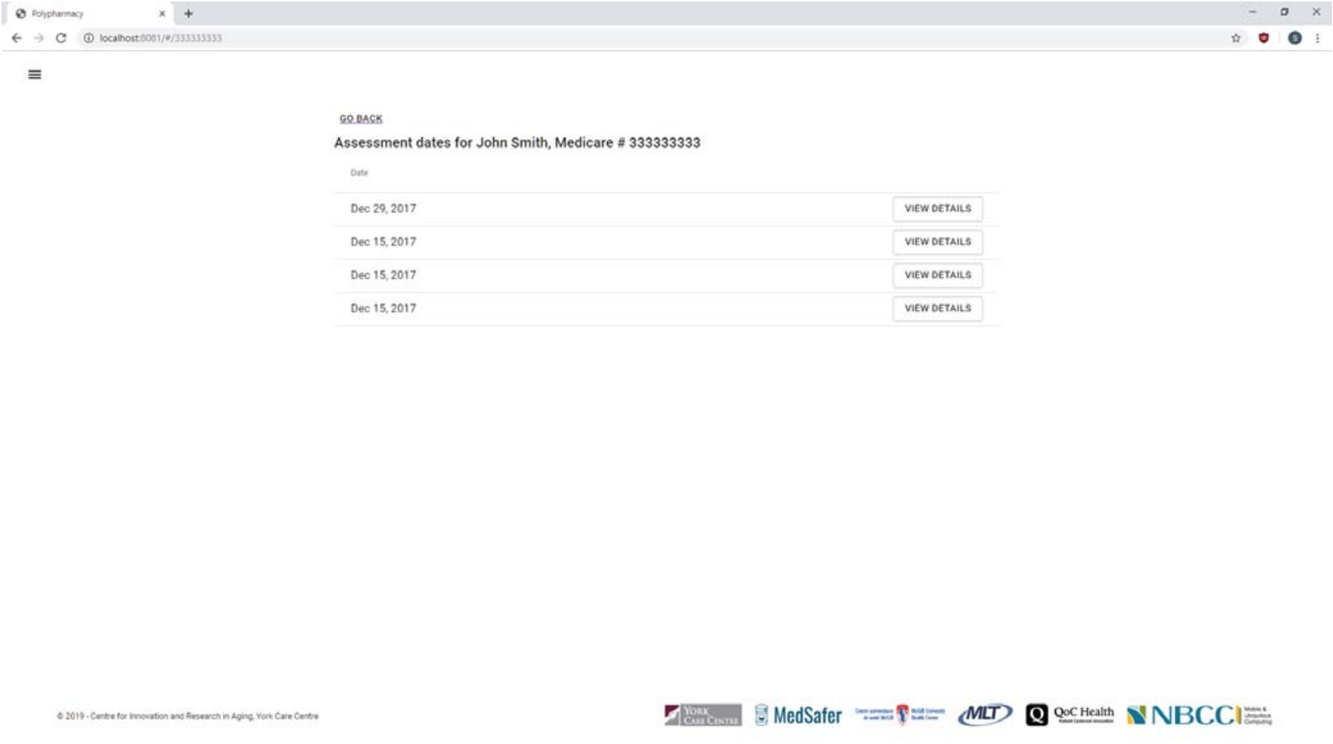

Figure 14. After a patient's *MedSafer Deprescribing Opportunities Reports*.

(4) After clicking on VIEW DETAILS, the *MedSafer Deprescribing Opportunities Report* will be made available for review as shown in the following view.

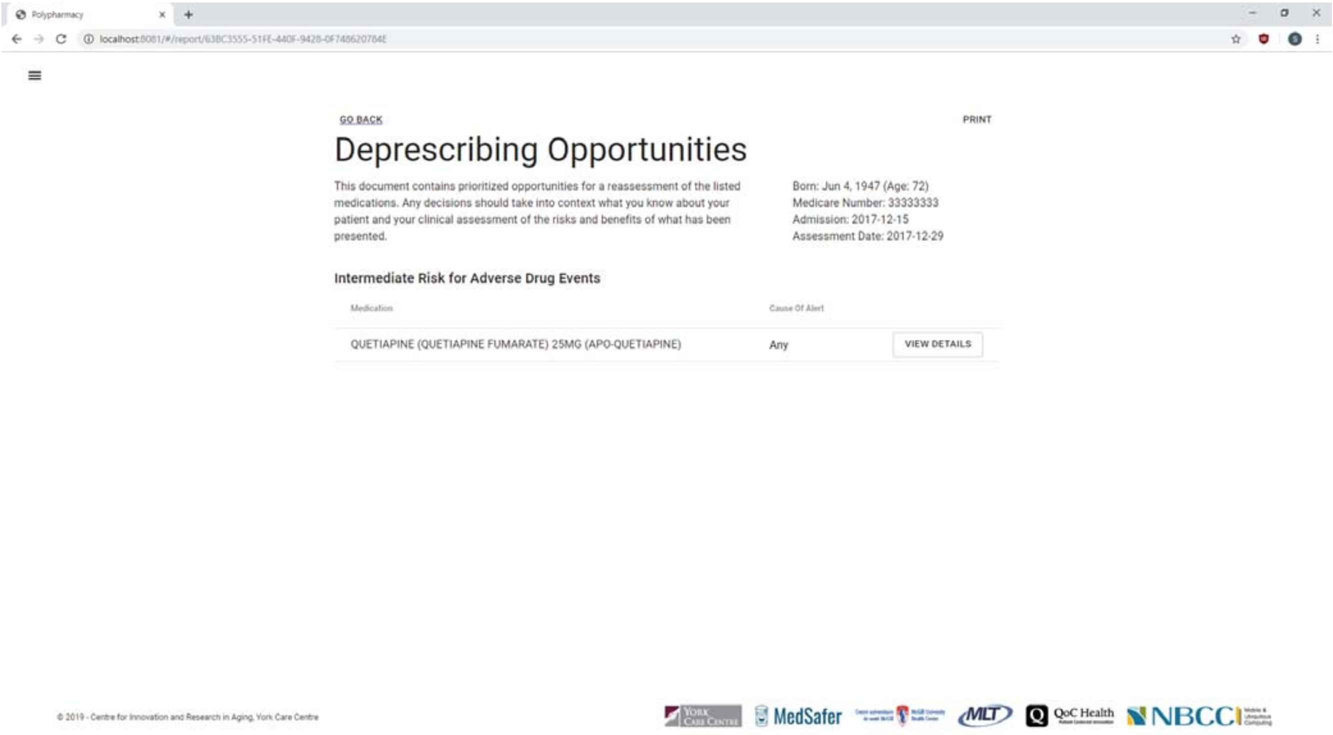

Figure 15. A *MedSafer Deprescribing Opportunities* report.

(5) Each *MedSafer Deprescribing Opportunities Report* offers additional details. Click the VIEW DETAILS button to view them. This is depicted in the following view.

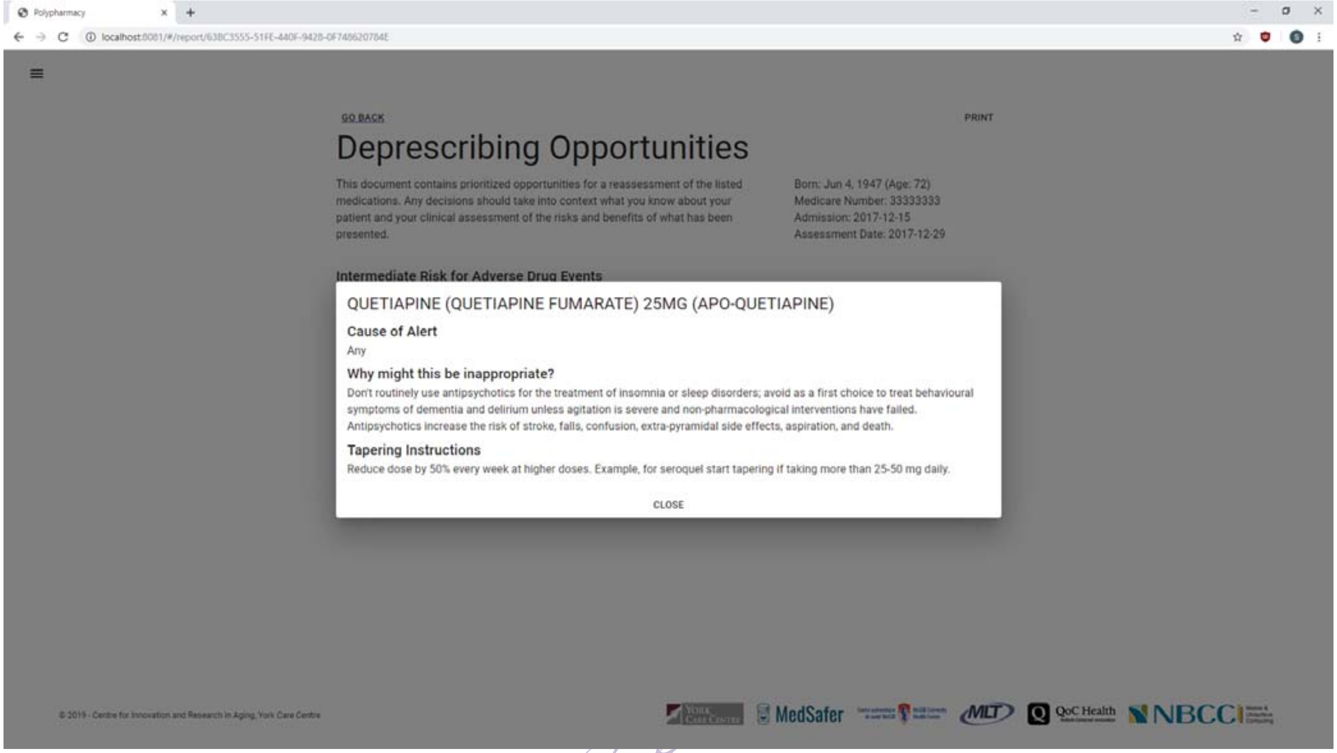

Figure 16. Viewing details within a *MedSafer Deprescribing Opportunities Report*.

(6) CLICK on the PRINT button at the top right of the view to save the *MedSafer Deprescribing Opportunities Report* to a PDF file or to send it to a printer. This is depicted in the following view.

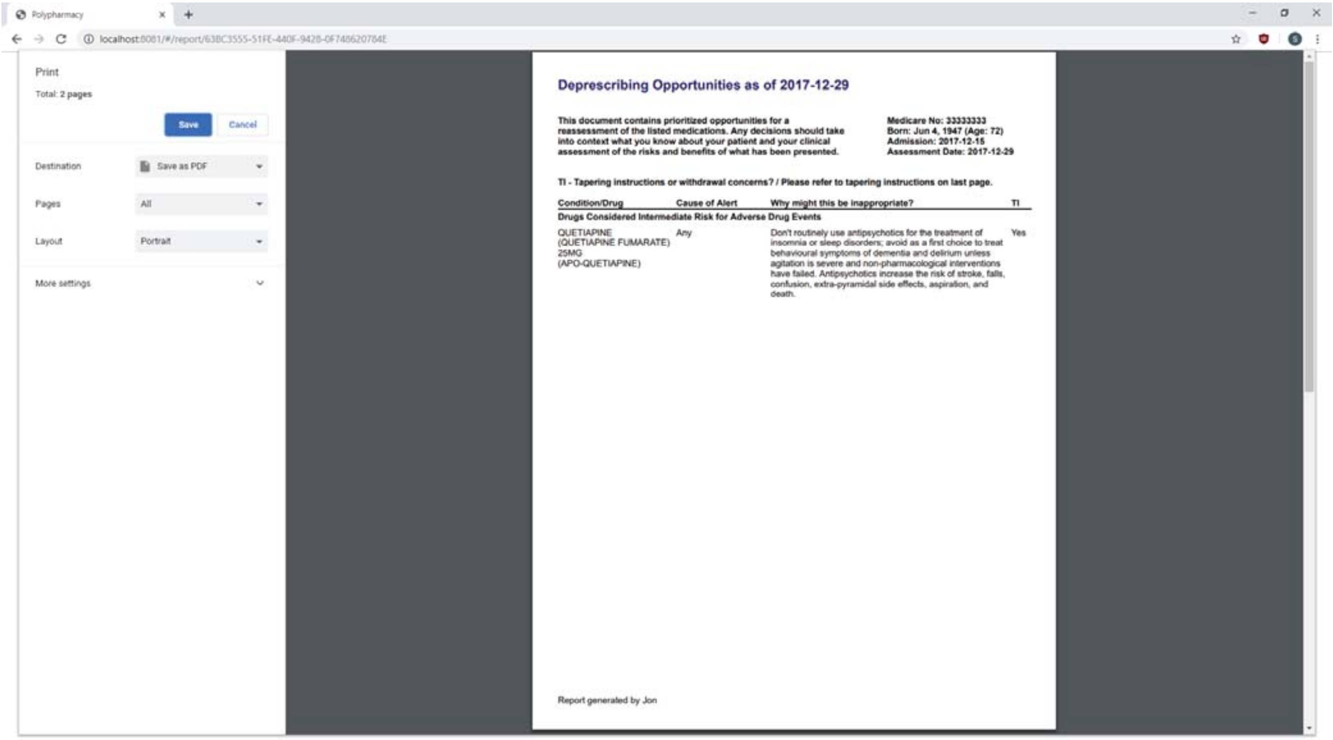

Figure 17. Print a *MedSafer Deprescribing Opportunities Report*.

DRAFT – CONFI
Cent

(7) The tasks explained above may be repeated any number of times for the same or different sets of LTCF reports.
(8) Log out of the *Clinical UI* of the *MedReviewRx App* when all tasks have been completed by opening the MENU on the left side of the view and then clicking *Log out.* This is depicted in the following view.

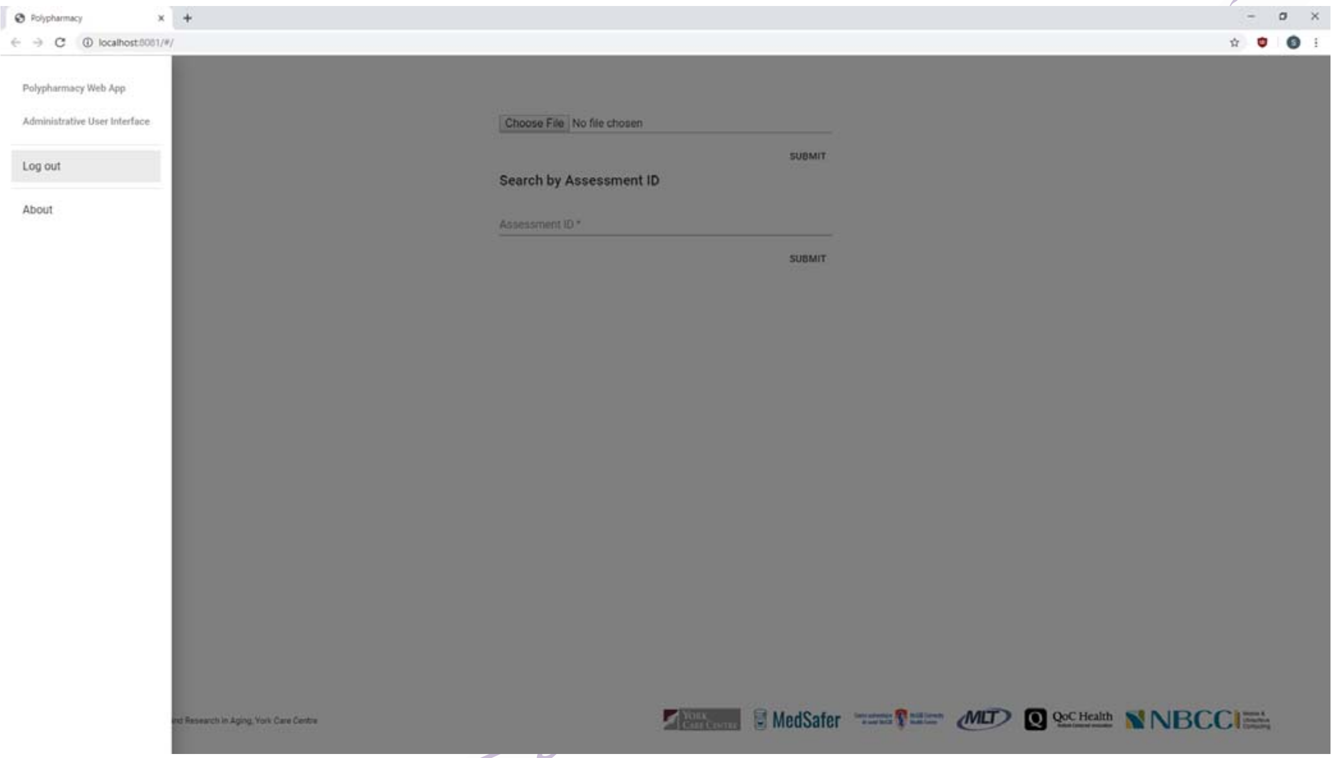

Figure 18. Logging out of the *MedReviewRx App*.

DRAFT – CO

(9) The following view will then be presented after you have logged out.

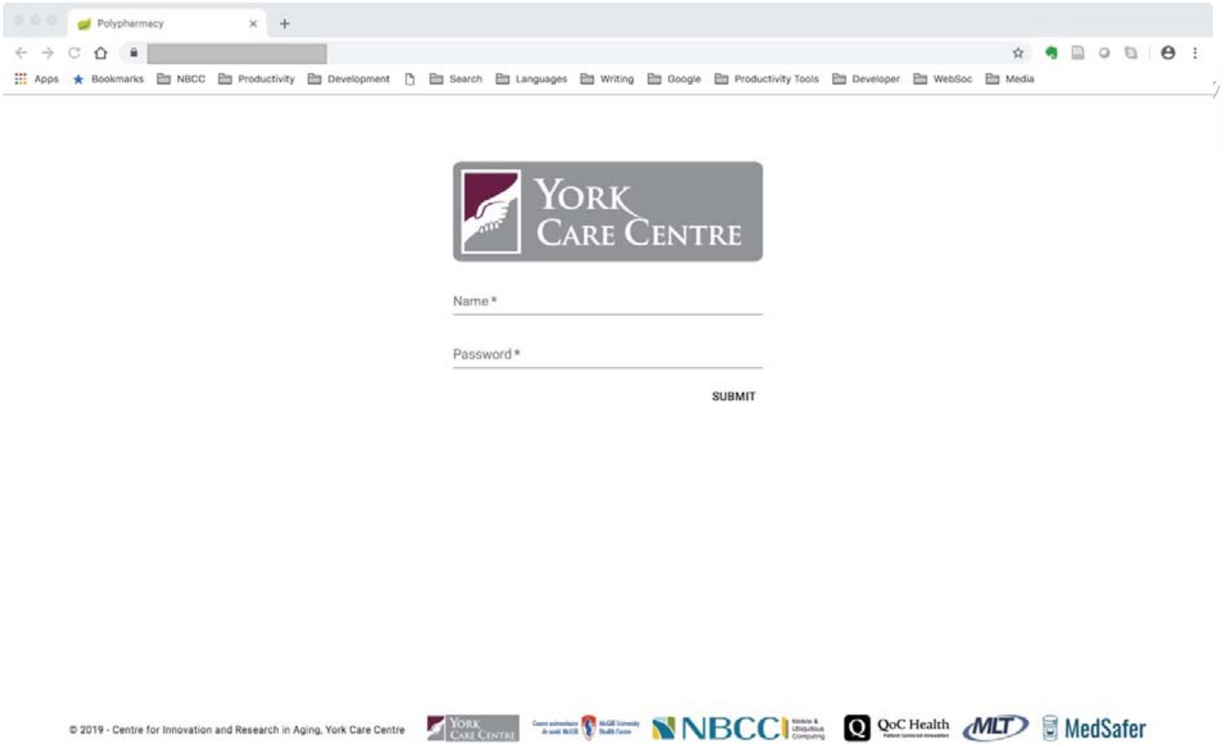

Figure 19. After logging out of the *MedReviewRx App*.

7 Help

Please contact *York Care Centre's Centre for Innovation and Research in Aging* if you have difficulty using the *MedReviewRx App*.

8 ACKNOWLEDGEMENTS

Copyright 2019 – Centre for Innovation and Research in Aging, York Care Centre.

The *Polypharmacy App* is a component of the *Polypharmacy System*, a software system that also includes the supporting *Polypharmacy Web Service*.

The Polypharmacy System simplifies the manual and tedious process of producing personalized deprescribing recommendations for each patient within a large care facility by integrating an existing source of electronic medication administration records with an electronic deprescribing tool.

The Polypharmacy System obtains deprescribing recommendations from the *MedSafer* electronic deprescribing system developed by Todd Campbell Lee, M.D. and Emily McDonald, M.D. of the Faculty of Medicine at McGill University.

The Polypharmacy System obtains HIPPA and PHIPA-compliant data security and privacy for the patient information it manages from a version of the *QoC Health* platform that was specially-adapted for this system by QoC Health Inc.

York Care Centre collects electronic medication administration data from each of its patients as part of a periodic assessment process that uses the interRAI Long-Term Care Facilities Assessment System (LTCF) standard. York Care Centre uses the *Momentum AMS* software application from *Momentum Healthware* to collect and manage these LTCF assessments. The Polypharmacy System uses the electronic medication administration data contained within LTCF records that have been exported from Momentum AMS as the bases of the requests that it makes to the MedSafer system for deprescribing recommendations.

The Polypharmacy System was conceived of by Kevin Harter, former CEO of York Care Centre.

Carole Goodine, BSc (Pharm), ACRP, Pharm D, and AGE-WELL/NBHRF Fellow of the Centre for Innovation and Research in Aging (CIRA) at York Care Centre led the project that developed the Polypharmacy System.

Missing Link Technologies Ltd. developed the software system for the Polypharmacy System.

The New Brunswick Community College – Centre for Mobile and Ubiquitous Computing (NBCC-MU) designed the software architecture and user interface for the Polypharmacy System and developed initial versions of the software.

Research, design, development and testing of the Polypharmacy System was supported by generous grants and support from the Centre for Aging + Brain Health Innovation, the New Brunswick Health Research Foundation, AGE-WELL NCE (Aging Gracefully across Environments using Technology to Support Wellness, Engagement and Long Life NCE Inc.), and the New Brunswick Innovation Foundation.

DRAFT – CONFIDENTIAL – York Care Centre CIRA

Base support for NBCC-MU is provided by the Natural Sciences and Engineering Research Council of Canada.

—

The MedSafer project has been supported by the following organizations: The McGill University Health Center Association of Physicians, The Canadian Frailty Network (CFN), Le Fonds de Recherche du Quebec - Sante (FRQS), McGill Clinical and Health Informatics (MCHI), Canadian Institutes of Health Research (CIHR).

LINKS

- Centre for Innovation and Research in Aging, York Care Centre | http://www.yorkcarecentre.ca
- MedSafer | https://www.medsafer.org
- Momentum Healthware| https://www.momentumhealthware.com
- QoC Health | https://qochealth.com
- Missing Link Technologies | http://mlt.ca
- New Brunswick Community College – Centre for Mobile and Ubiquitous Computing | https://nbcc.ca/mobi

DRAFT – CONFIDENTIAL – York Care Centre CIRA

9 Appendix – Exporting Records from Momentum in a Format Compatible with the Polypharmacy System

LTCF records should be exported according to the following specifications so that they can be imported into the Polypharmacy System:

1. Export LTCF records from Momentum.
2. Save as CSV.
3. Specify comma delimited when saving the LTCF records.

DRAFT – CONFIDENTIAL – York Care Centre CIRA

How to use the InterRAI IRRS LTCF Data Extract Report

The interRAI IRRS LTCF Data Extract Report was developed for the MedReviewRx research project.

This report will allow data to be extracted from electronic interRAI LTCF assessments and loaded into the MedReviewRx system.

To use the report:

1) Log into Momentum
2) Open the Report menu

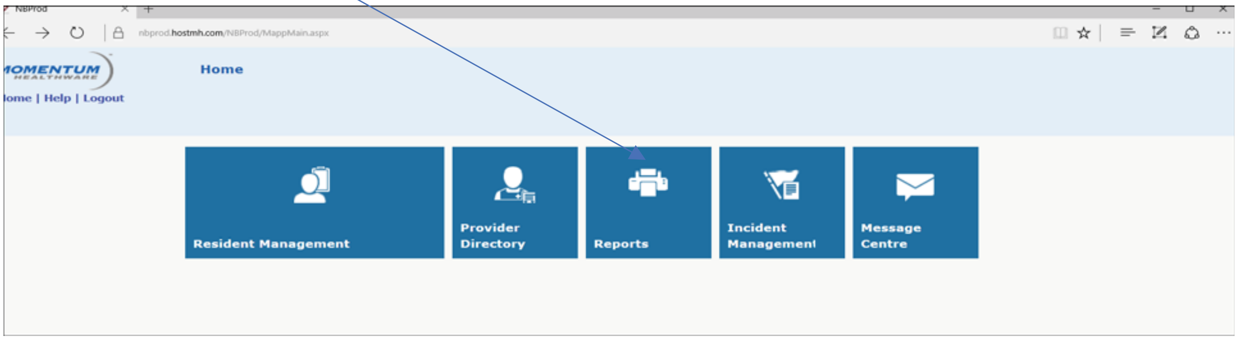

3) Find the InterRAI IRRS LTCF Data Extract report

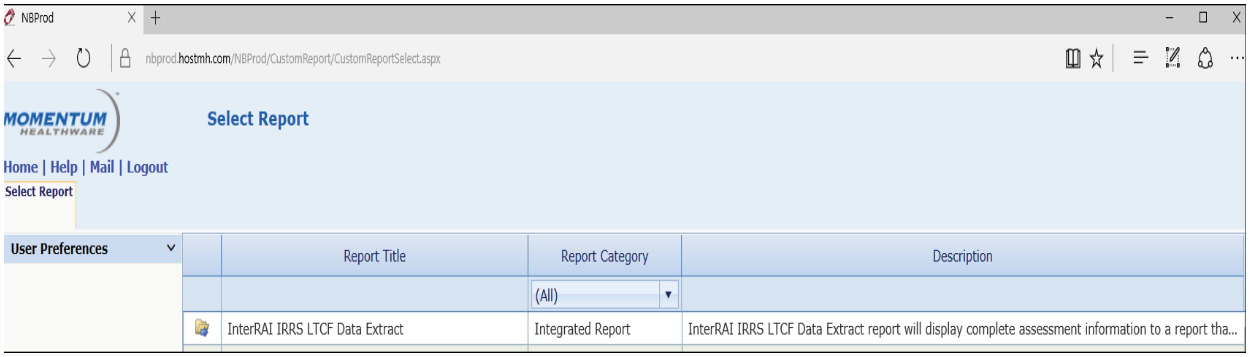

4) Open the InterRAI IRRS LTCF Data Extract report
   - Set the Record status to “All”
   - Enter a start date and stop date (for weekly downloads start on a Monday and end on a Sunday. For monthly downloads Start the first of the month and end the end of the month).
   - Make sure to set the View Report to “CSV”
   - Click on View report button

Appendix G – Instructions on how to use the Momentum extract report

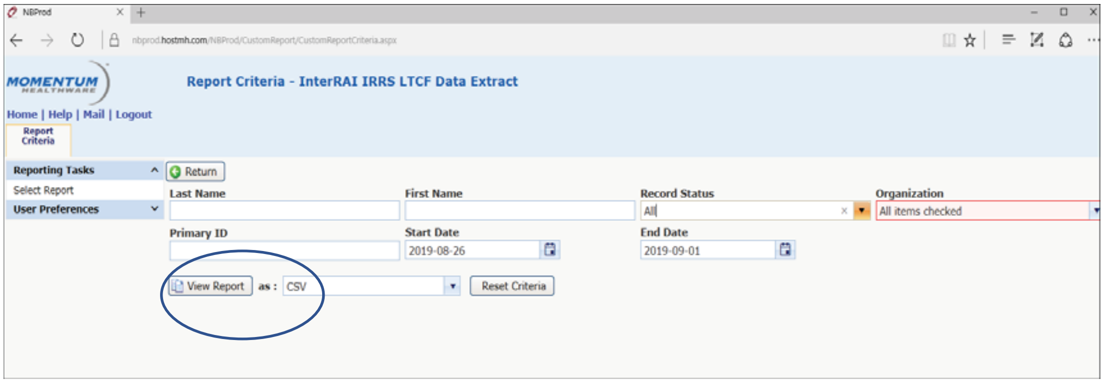

5) The report will generate, and you will see the following screen:

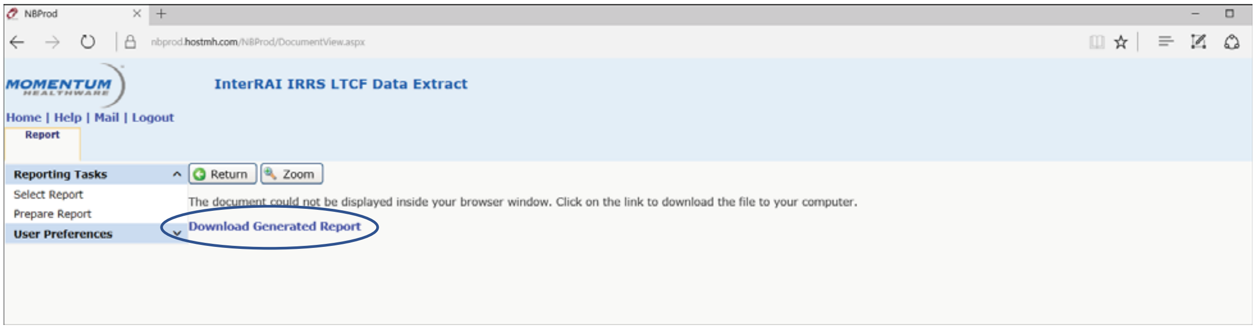

6) Click on "Download Generated Report"

You will be prompted to save the report.

7) Click "Save as"

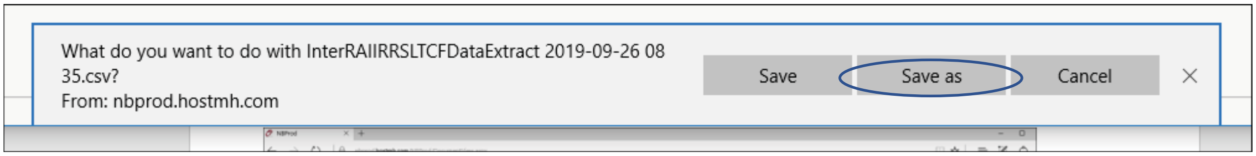

8) Save files in a designated study site data folder for Momentum export reports on your secure password protected system
9) Indicate the dates contained in the file when renaming the file.

Appendix J: Sample study FAQ announcement

MedReviewRx Project: FAQ Communication Template for Public Announcements

RE: MedReviewRx Project

<insert nursing home name> is taking part in a research study to test a new computer system called MedReviewRx. MedReviewRx helps doctors, nurse practitioners and pharmacists review medicine to make sure it is safe for older adults who live in a nursing home.

Why is this project important?

Almost 2/3 of older adults who live in a nursing home take 5 or more medicines. Up to 40% of older adults who live in a nursing home may take a medicine that is harmful or not needed. Harmful medicine makes it more likely that an older adult will fall, have memory problems, need to visit the hospital or even die.

How can we make sure medicine is safe?

A review of medicine lists to look for harmful medicine and medicine that may not be needed can improve safety. The process of reducing or stopping medicine is called deprescribing. Deprescribing is good patient care. Deprescribing takes time, needs resources and needs expert information.

How does MedReviewRx help?

MedReviewRx sends a person's medical history and medication list to a computer program called MedSafer. MedSafer analyses the information and makes a report which lists medicine that may be harmful and medicine that may not be needed. MedReviewRx allows health care providers to view MedSafer reports for older adults they care for.

What are the goals of this project?

The goal of this project is to use MedReviewRx in a small number of New Brunswick nursing homes to see how it works and what effect it has on the medicine used by older adults in nursing homes.

Will medications be automatically changed?

No, deprescribing is planned. Changing medicine is done by a doctor or nurse practitioner with input from the older adult or their family. There may be reasons to continue taking certain medicine or reasons why close supervision is needed.

Doctors and nurse practitioners do not need to stop medicine listed in MedReviewRx. They are asked to read the information and consider if the information make sense for each person based on their medical history, preferences and goals.

Who has approved this research?

This study has been reviewed and approved by the Horizon Health Network Research and Ethics Board. The study approval number is <insert>

Who is funding this project?
