# Supplementary material for: Electronic Decision Support for Deprescribing in Older Adults Living in Long-Term Care: A Stepped-Wedge Cluster Randomized Trial
Source: JAMA Netw Open. 2025 May 30;8(5):e2512931. doi: 10.1001/jamanetworkopen.2025.12931 (PMC12125643; doi:10.1001/jamanetworkopen.2025.12931)
Supplement: Supplement 2. — eAppendix 1. Examples of Deprescribing Opportunities Generated by MedSafer With App Screenshots eMethods. Supplemental eMethods eTable 1. Adjusted Odds of Deprescribing One or More Potentially Inappropriate Medications eTable 2. Adjusted Odds of Restraints Use eTable 3. Adjusted Odds of Having a Fall eAppendix 2. Description of the Deprescribing App Data Privacy and Security [file jamanetwopen-e2512931-s002.pdf]

Supplemental Online Content

McDonald EG, Estey JL, Davenport C, et al. Electronic decision support for deprescribing in older adults living in long-term care: a stepped-wedge cluster randomized trial. *JAMA Netw Open*. 2025;8(5):e2512931. doi:10.1001/jamanetworkopen.2025.12931

eAppendix 1. Examples of Deprescribing Opportunities Generated by MedSafer With App Screenshots
eMethods. Supplemental eMethods

eTable 1. Adjusted Odds of Deprescribing One or More Potentially Inappropriate Medications
eTable 2. Adjusted Odds of Having a Fall
eTable 3. Adjusted Odds of Restraints Use
eAppendix 2. Description of the Deprescribing App Data Privacy and Security

This supplemental material has been provided by the authors to give readers additional information about their work.

© 2025 McDonald EG et al. *JAMA Network Open*.

eAppendix 1. Examples of Deprescribing Opportunities Generated by MedSafer With App Screenshots

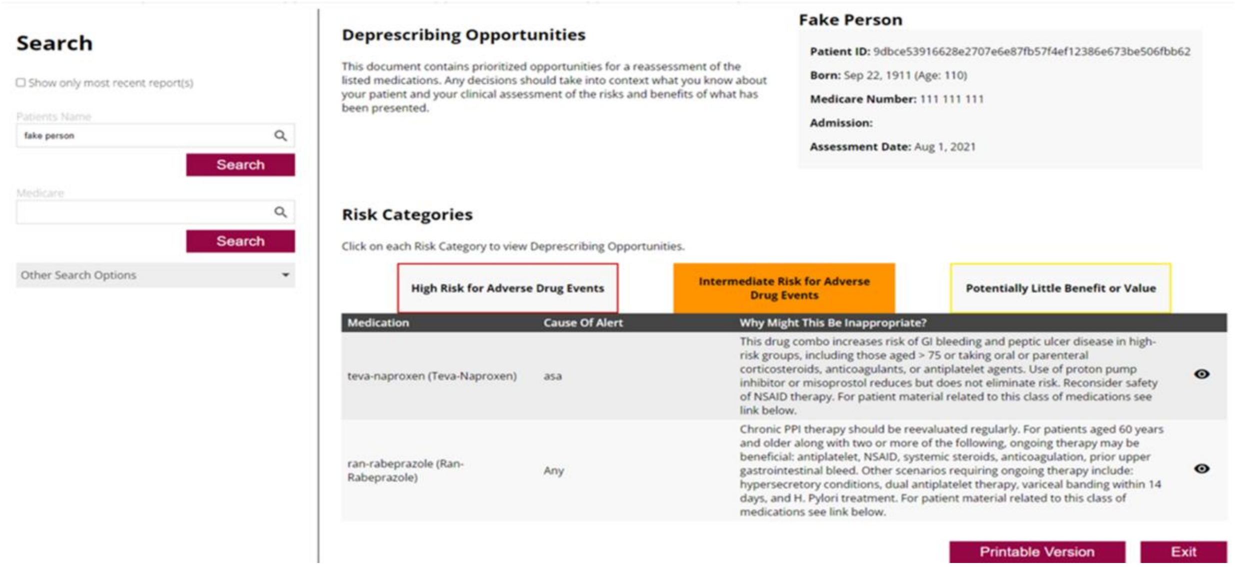

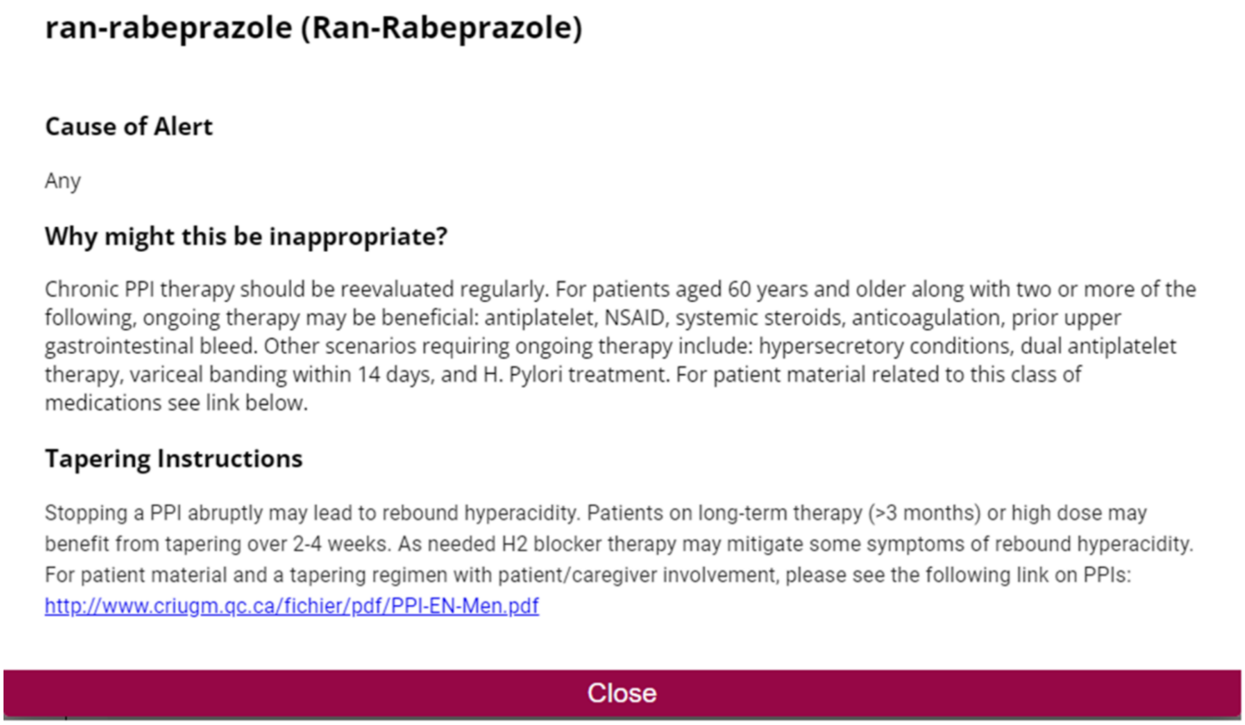

© 2025 McDonald EG et al. *JAMA Network Open*.

MedReviewRx

Deprescribing Opportunities as of 2021-08-01T00:00:00.000

This document contains prioritized opportunities for a reassessment of the listed medications. Any decisions should take into context what you know about your patient and your clinical assessment of the risks and benefits of what has been presented.

Fake Person
Medicare No: 111 111 111
Born: Sep 22, 1911 (Age: 110)
Admission:
Assessment Date: 2021-08-01T00:00:00.000

TI - Tapering instructions or withdrawal concerns? / Please refer to tapering instructions on last page.

Condition/Drug	Cause of Alert	Why might this be inappropriate?	TI
**Drugs Considered Intermediate Risk for Adverse Drug Events**			
teva-naproxen (Teva-Naproxen)	asa	This drug combo increases risk of GI bleeding and peptic ulcer disease in high-risk groups, including those aged > 75 or taking oral or parenteral corticosteroids, anticoagulants, or antiplatelet agents. Use of proton pump inhibitor or misoprostol reduces but does not eliminate risk. Reconsider safety of NSAID therapy. For patient material related to this class of medications see link below.	Yes
ran-rabeprazole (Ran-Rabeprazole)	Any	Chronic PPI therapy should be reevaluated regularly. For patients aged 60 years and older along with two or more of the following, ongoing therapy may be beneficial: antiplatelet, NSAID, systemic steroids, anticoagulation, prior upper gastrointestinal bleed. Other scenarios requiring ongoing therapy include: hypersecretory conditions, dual antiplatelet therapy, variceal banding within 14 days, and H. Pylori treatment. For patient material related to this class of medications see link below.	Yes

Report generated by Cody Davenport

© 2025 McDonald EG et al. *JAMA Network Open*.

Tapering Instructions

teva-naproxen (Teva-Naproxen) Cause of Alert: asa

For patient material on NSAIDs:
http://www.criugm.qc.ca/fichier/pdf/Empower_NSAIDS_EN.pdf

ran-rabeprazole (Ran-Rabeprazole) Cause of Alert: Any

Stopping a PPI abruptly may lead to rebound hyperacidity. Patients on long-term therapy (>3 months) or high dose may benefit from tapering over 2-4 weeks. As needed H2 blocker therapy may mitigate some symptoms of rebound hyperacidity. For patient material and a tapering regimen with patient/caregiver involvement, please see the following link on PPIs:
http://www.criugm.qc.ca/fichier/pdf/PPI-EN-Men.pdf

Prescriber Signature ______________________________________ Date_________________

Comments/feedback:

____

____

Report generated by Cody Davenport

© 2025 McDonald EG et al. *JAMA Network Open*.

eMethods. Supplemental eMethods

Examples of potentially inappropriate medications by category:

Little added value:
- Docusate
- Multiple daily doses of iron
- Multiple daily doses of calcium
- Vitamin D (and other vitamins) and a limited life prognosis
- Lipid lowering drugs and a limited life prognosis

Intermediate risk (clinician must weigh the individual harm to benefit ratio):
- Proton pump inhibitors
- Iron pills in the presence of constipation
- Corticosteroids for the treatment of osteoarthritis
- Corticosteroids for the treatment of chronic obstructive pulmonary disease
- Ipratropium and glaucoma
- Ranitidine and history of delirium
- Oral bisphosphonates and history of gastroesophageal reflux disease
- Estradiol and a history of breast cancer
- Estradiol and a history of venous thromboembolism
- Anticholinergics and urinary retention or benign prostatic hypertrophy
- Donepezil or memantine with predisposing condition that could worsen (e.g., recurrent falls; bradycardia; orthostatic hypotension)
- Citalopram and hyponatremia

High risk (harms outweigh benefits for most residents):
- Sleeping pills
- Antipsychotics as first line for agitation or sleep
- Combination blood thinners beyond six months (e.g., aspirin and clopidogrel)
- Opioids for chronic non-cancer pain
- Glyburide
- Any anticholinergic with a predisposing condition that could worsen (e.g., delirium, dementia)
- Gabapentinoids
- Combination anticholinergics
- Sulfonylureas and a history of hypoglycemia
- First generation antipsychotics and a predisposing condition that could worsen (e.g., Parkinsons, urinary retention, benign prostatic hypertrophy)
- Diabetes treatments and a tightly controlled hemoglobin a1c (e.g., <7.5%)

© 2025 McDonald EG et al. *JAMA Network Open*.

Examples of drugs with tapering instructions:

- Proton pump inhibitors
- Corticosteroids
- Antipsychotics
- Benzodiazepines
- Trazodone
- Z-drugs (e.g., zopiclone)
- Antidepressants (e.g., selective serotonin reuptake inhibitors; amitriptyline; bupropion)
- Donepezil and memantine
- Isosorbide dinitrate
- Opioids

Tapering instructions were derived from medstopper.com (freely available Canadian software); for drugs where tapering was not required, this was also indicated (e.g., “no tapering required” was presented to the user).

© 2025 McDonald EG et al. *JAMA Network Open*.

Handling of repeated measurements:

Due to the nature of the study design, residents could contribute data to both the intervention and control phases. For example, resident A could meet the primary outcome in the control phase if they had 1 or more PIMs deprescribed following a control phase medication review. Resident A could also contribute to the primary outcome if they had one or more PIMs deprescribed during the intervention phase. Resident A only contributed data to the control phase If they died or were transferred prior to the intervention phase. If a resident had no medication deprescribed in the control phase, and only in the intervention phase, then they contributed data to the primary outcome in the intervention and were included in the denominator in both the control and intervention phase.

© 2025 McDonald EG et al. *JAMA Network Open*.

Canadian and New Brunswick (NB) long term care context:

In Canada, the type of costs covered in nursing homes vary across the country. Ownership of nursing homes is a mixture of public and privately owned homes (which can be for profit or not-for-profit).

There are no privately owned nursing homes in NB. In 2021, there were 70 nursing homes in NB, 14% were private for-profit and 86% were private not-for-profit ownership. (CIHI, 2021) The number of NB nursing homes has increased to 77 and continued increases are expected due to the aging population. Nursing homes are intended for individuals who are medically stable and require 24-hour nursing care. NH admission is voluntary however, nursing home admissions must be approved by the NB department of Social Development and there is a cost for room and board. Those who cannot afford these costs can apply for a subsidy. Most individuals living in a NB nursing home are eligible for the New Brunswick Prescription Drug Program (NBPDP) and receive medications approved under NBPDP at no cost. Medications not approved by NBPDP must be paid for by the individual.

Canadian Institute for Health Information. Long-term care homes in Canada: How many and who owns them?. Accessed March 9, 2025.

All New Brunswick Nursing Homes (NBNHs) contract a physician who acts as the House Physician (medical director) who provides care to residents as well as clinical and administrative advice to the staff, administrator and board of the nursing home. Larger NB nursing homes will have additional physicians under contract and a small number of NBNHs also have nurse practitioners (NPs) provided by the Department of Health through a Regional Health Authority. An attending physician is appointed to be responsible for the care of each resident. Contracted physicians must visit the NH on a weekly basis and be on-call for urgent medical services for residents under their care. NPs work on-site Monday through Friday under a collaborative practice model with a physician. Only one NH in our study had NPs onsite.

© 2025 McDonald EG et al. *JAMA Network Open*.

eTable 1. Adjusted Odds of Deprescribing One or More Potentially Inappropriate Medications

	Odds ratio	95% CI
Intervention	1.58*	[1.07; 2.34]
Period	1.68**	[1.41; 2.01]
Number of PIM	1.09**	[1.05; 1.14]
Age, per year	1.00	[0.98; 1.01]
Male sex	0.84	[0.64; 1.10]
English language	1.45	[0.92; 2.27]
	Random variance	ICC
ICC for Cluster	0.013	0.40%
ICC for Sites nested in Cluster	0.082	2.42%
ICC for participants nested in Sites	0.104	3.06%

Adjusted for site clustering and repeated measurements, *significant at 95%, **significant at 99%

ICC=intracluster correlation

PIM=potentially inappropriate medication

© 2025 McDonald EG et al. *JAMA Network Open*.

eTable 2: Adjusted Odds of Having a Fall

	Odds ratio	95% CI
Intervention	1.77**	[1.15; 2.71]
Period	0.92	[0.77; 1.11]
Number of PIM	1.14**	[1.08; 1.20]
Age, per year	1.01	[0.99; 1.02]
Male sex	1.51*	[1.05; 2.20]
English language	1.77	[0.99; 3.16]
	Random variance	ICC
ICC for Cluster	0.071	2.11%
ICC for Sites nested in Cluster	0.189	5.43%
ICC for participants nested in Sites	2.228	40.40%

Adjusted for site clustering and repeated measurements, *significant at 95%, **significant at 99%

ICC=intracluster correlation
PIM=potentially inappropriate medication

eTable 3. Adjusted Odds of Restraint Use

	Odds ratio	95% CI
Intervention	1.95	[0.52; 7.37]
Period	2.91**	[1.65; 5.14]
Number of PIM	1.12	[0.97; 1.28]
Age, per year	1.01	[0.94; 1.10]
Male sex	0.88	[0.18; 4.42]
English language	1.24	[0.10; 15.44]
	Random variance	ICC
ICC for Cluster	0.099	2.92%
ICC for Sites nested in Cluster	0.322	8.91%
ICC for participants nested in Sites	309.1	98.94%

Adjusted for site clustering and repeated measurements, *significant at 95%, **significant at 99%

ICC=intracluster correlation
PIM=potentially inappropriate medication

© 2025 McDonald EG et al. *JAMA Network Open*.

eAppendix 2. Description of the Deprescribing App Data Privacy and Security

The App addressed provincial data privacy and security requirements. The customized extract report was written to permit only patient demographics, medical conditions, medication information and safety monitoring data to be downloaded from Momentum Software interRAI assessment data and uploaded into the Polypharmacy App in batches by a designated person at each study site prior to the quarterly medication review. Identifiable patient information was removed, and a unique identifier was assigned to each person's data set. Anonymized data sets were then encrypted and securely transmitted to MedSafer for analysis. MedSafer was unable to identify patients; the unique identifier allowed the Polypharmacy and MedSafer applications to anonymously communicate about specific patients through the API. The Polypharmacy App system was partitioned into three security domains, designed to isolate any breaches that might occur to one domain. Secure data communication protocols were used for all interactions between domains. The Polypharmacy App was built with funding from the Government of New Brunswick and the Public Health Agency of Canada's Healthy Seniors Pilot Project. A full detail of security of the App can be under the section: Security safeguards in the MedReviewRX system.

© 2025 McDonald EG et al. *JAMA Network Open*.
