# Supplementary material for: Electronic Decision Support for Deprescribing in Older Adults Living in Long-Term Care: A Stepped-Wedge Cluster Randomized Trial
Source: JAMA Netw Open. 2025 May 30;8(5):e2512931. doi: 10.1001/jamanetworkopen.2025.12931 (PMC12125643; doi:10.1001/jamanetworkopen.2025.12931)
Supplement: Supplement 3. — Data Sharing Statement [file jamanetwopen-e2512931-s003.pdf]

Data Sharing Statement

McDonald. Electronic Decision Support for Deprescribing in Older Adults Living in Long-Term Care. *JAMA Netw Open*. Published May 30, 2025. doi:10.1001/jamanetworkopen.2025.12931

Data
Additional Information: NCT04762303
Data available: No

Additional Information
Explanation for why data not available: Data will be shared upon reasonable request; an inter-institutional and data sharing agreement are needed.
